# From anonymity to stardom: history of nontuberculous mycobacterial disease in humans

**DOI:** 10.3389/fcimb.2025.1717909

**Published:** 2026-02-23

**Authors:** Surendra Kumar Sharma, Vishwanath Upadhyay, Alladi Mohan

**Affiliations:** 1 Faculty of Medicine, Datta Meghe Institute of Medical Sciences (Deemed to be University), Wardha, Maharashtra, India; 2 Department of Molecular Medicine, Jamia Hamdard (Deemed to be University), New Delhi, India; 3 Senior Scientist Fellow, National Academy of Sciences, India (NASI), Prayagraj, Uttar Pradesh, India; 4 Department of Life Science, Sharda University, Noida, Uttar Pradesh, India; 5 Department of Medicine, Sri Venkateswara Institute of Medical Sciences, Tirupati, Andhra Pradesh, India

**Keywords:** emerging respiratory pathogens, *Mycobacterium abscessus* complex, *Mycobacterium avium* complex (MAC), *Mycobacterium kansasii*, nontuberculous mycobacteria (NTM), NTM disease history, pulmonary and extrapulmonary NTM disease

## Abstract

Until recently, nontuberculous mycobacteria (NTM) were not considered as human pathogens. Their nomenclature has evolved over several years, until 1971 when finally named as NTM, which is universally accepted now. Because of continuously evolving diagnostic methods, several new species/subspecies of NTM were identified. Presently, nearly 200 NTM species have been reported. Similar to tuberculosis (TB), NTM are known to involve both pulmonary and extrapulmonary organs. Diagnosis of NTM disease is quite cumbersome and is primarily based on their growth characteristics on solid and liquid cultures. Biochemical testing was the mainstay of NTM diagnosis and species identification in the past, which was often erroneous. Recently, molecular tests like line probe assay, targeted, and whole genome sequencing have become available for rapid and accurate diagnosis. Isolation and identification of NTM species/subspecies alone do not warrant treatment. After ascertaining clinical relevance, virulence of NTM species, and evidence for clinical and radiological progression, the decision to administer treatment is taken. Multiple drugs are often administered for 12 months after sputum culture conversion, except in *M. kansasii*, and *M. szulgai*, where this constitutes the total treatment duration, with careful follow‑up for relapse and exogenous new infection. The present review traces the history, evolution of classification of NTM, strides made in the diagnosis and treatment of NTM disease. By integrating these historical lessons on taxonomy, culture phenotypes, genotypes and diagnostic pitfalls with contemporary molecular tools and species/subspecies specific treatment regimens, current practice enables more precise, timely, and patient−centered management of NTM disease.

## 1 Introduction

Nontuberculous mycobacteria (NTM) are mycobacteria other than *Mycobacterium tuberculosis* (the causative agent of tuberculosis [TB]) and *Mycobacterium leprae* (the causative agent of leprosy). These mycobacteria have also been referred by several other names, including ‘paratubercle bacilli’, ‘pseudotubercle bacilli’, ‘tuberculoid bacilli’, ‘unclassified’, ‘anonymous’, ‘atypical’, ‘environmental’, ‘opportunistic’, and mycobacteria other than *M. tuberculosis* complex (MOTT) (Turenne, 2019). Though NTM had existed for a long time, because of similarity with *Mtb* and lack of clarity about their nomenclature, these were neglected. In 1981, with the advent of human immunodeficiency virus (HIV) infection, these organisms came into limelight and importance of localised and disseminated NTM disease was recognized.

Historically the first account of these ‘atypical’ mycobacteria can be traced back to the 19^th^ century, when “tuberculosis “in chickens was described in 1868 (Nontuberculous mycobacteria (overview)). By 1890, it was discovered in the laboratory that the organism was different from *M. tuberculosis* and later was identified to be *Mycobacterium avium* and it was realized by then that these organisms did not cause disease in humans (Nontuberculous mycobacteria (overview)). In 1926, *M. marinum* infection in salt water fish was reported (Aronson, 1926). Later in early 1930s, atypical mycobacteria were recognized to cause diseases in humans (Branch, 1933). In 1938, da Costa Cruz (1938) described *M. fortuitum* (da Costa Cruz, 1938). In 1943, the first case of atypical mycobacteria-associated pulmonary disease due to *M. avium* comple*x* (MAC) was described in a patient with underlying silicosis (Runyon, 1959). Soon, it was realized that the atypical mycobacteria-associated lung disease did not respond to anti-TB drugs (Nontuberculous mycobacteria (overview)).

The history of NTM is marked by profound knowledge gaps, both globally and specifically in India. Historically, diagnostic limitations have been a primary obstacle; reliance on smear microscopy for TB diagnosis meant countless NTM cases were likely misidentified as “smear-positive, culture-negative TB,” making their true prevalence fundamentally unknowable.

With the exception of Buruli ulcer in specific regions of the world globally (7), NTM was never subjected to systematic international surveillance, leaving vast regions of Asia, Africa, and South America as historical blind spots. Early data often failed to differentiate between NTM species, rendering it uninterpretable. A central dogma—that NTM was only acquired from the environment—has recently been challenged, raising the critical and unanswered question of whether unrecognized human-to-human transmission has always occurred. This is compounded by a major therapeutic gap, as the development of drugs specifically for NTM has seldom been done.

The following description provides an account of the evolution of historical aspects regarding NTM.

## 2 Historical account: global perspective

**Figure 1:**
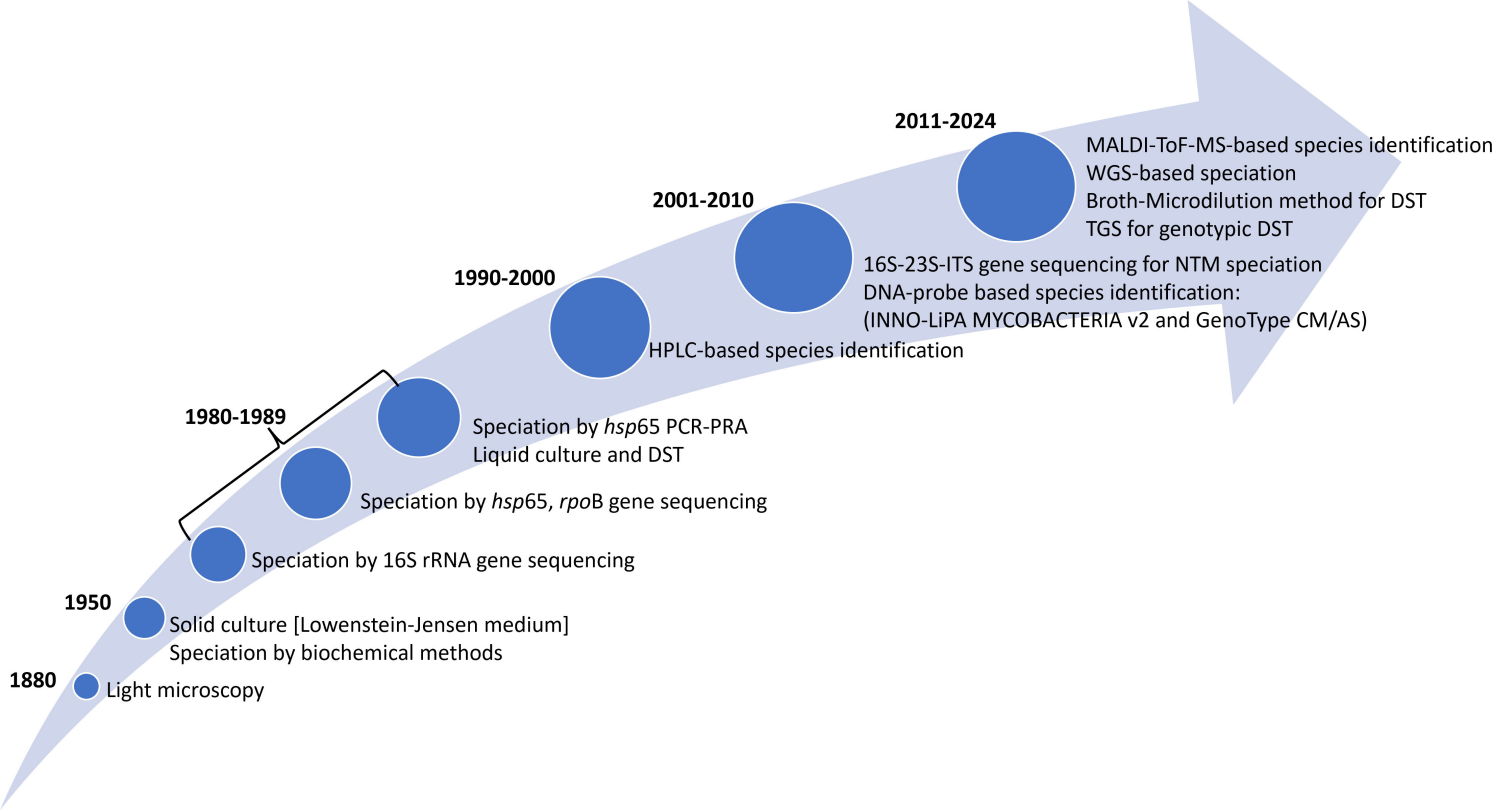
Timeline of various laboratory techniques for NTM isolation and identification of its species. rRNA, ribosomal ribonucleic acid; DNA, deoxyribonucleic acid; DST, drug-susceptibility testing; HPLC, high performance liquid chromatography; PCR-PRA, polymerase chain reaction restriction fragment length polymorphism; CM/AS, common mycobacteria/additional species; MALDI TOF MS, matrix assisted laser desorption time of flight mass spectrometry; ITS, internal transcribed spacer; WGS, whole genome sequencing; TGS, targeted gene sequencing.

**Figure 2:**
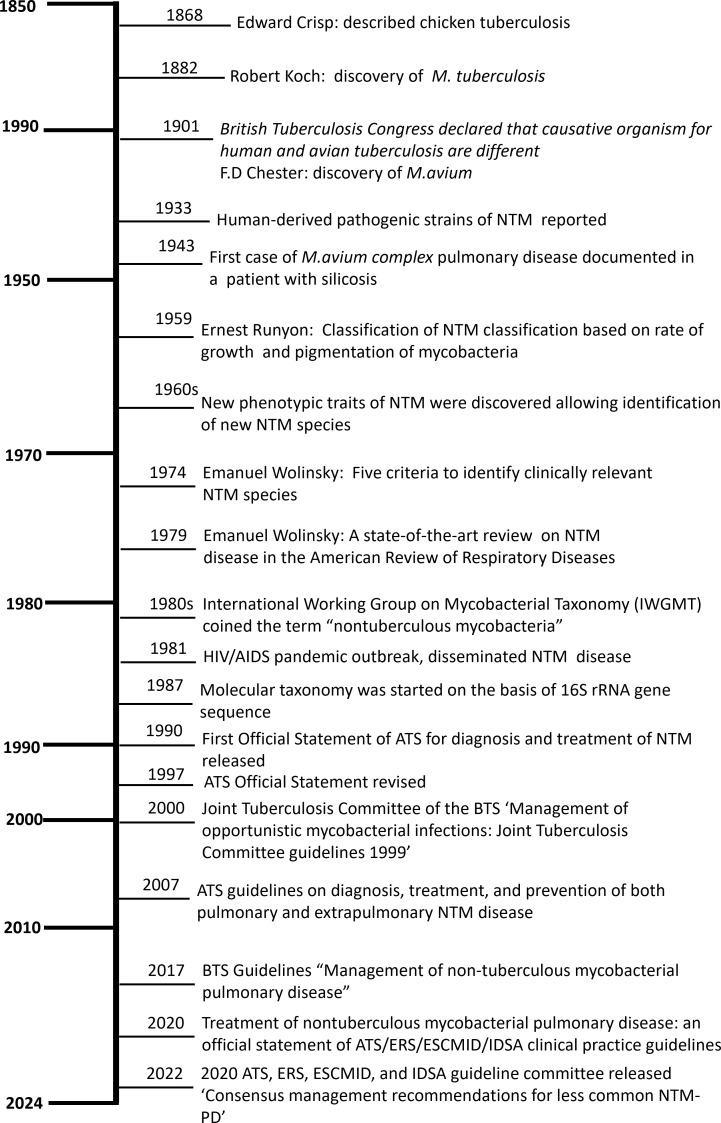
History of nontuberculous mycobacteria: global perspective. NTM, nontuberculous mycobacteria; IWGMT, International Working Group on Mycobacterial Taxonomy; HIV, human immunodeficiency virus; AIDS, acquired immunodeficiency syndrome; rRNA, ribosomal ribonucleic acid; ATS, American Thoracic Society; BTS, British Thoracic Society; ERS, European respiratory Society; ESCMID, European Society of Clinical Microbiology and Infectious Diseases; IDSA, Infectious Diseases Society of America.

A narrative of various historic events leading to our present knowledge regarding these atypical mycobacteria is detailed in **Table 1**, **Figures 1**, **2** (Alvarez and Tavel, 1885; Marzinowski, 1900; Ophüls, 1904; Cobbett, 1918; Wilson, 1925; Aronson, 1926; Branch, 1933; da Costa Cruz, 1938; MacCallum et al., 1948; Cuttino and McCabe, 1949; Norden and Linell, 1951; Tarshis and Frisch, 1952a; Tarshis and Frisch, 1952b; Tarshis and Frisch, 1952c; Buhler and Pollak, 1953; Gibson, 1953; Karlson and Feldman, 1953; Lominski and Harper, 1953; Moore and Frerichs, 1953; Palmer, 1953; Timpe and Runyon, 1954; Runyon, 1959; Runyon, 1959; Anonymous, 1990; Anonymous, 1997; Subcommittee of the Joint Tuberculosis Committee of the British Thoracic Society. Management of opportunist mycobacterial infections: Joint Tuberculosis Committee Guidelines 1999, 2000; Griffith et al., 2007; Haworth et al., 2017; Daley et al., 2020; Lange et al., 2022; Ravindran et al., 2025). For a long time, clinical isolates of these atypical mycobacteria from pulmonary specimens were often considered as contaminants without any clinical significance (Runyon, 1959). During the 1940s and 1950s, these acid-fast organisms were considered as saprophytes, unlike *Mtb*, they were not considered pathogenic in guinea pigs (Runyon, 1959) as per the dogmatic belief that acid-fast bacilli (AFB) were unable to produce disease in guinea pigs was *ipso facto* a saprophyte (Runyon, 1959; Cummins and Williams, 1933). Repeated isolation of AFB from sputum specimens, on culture and later on isolation from the resected lung specimen from the same patient, that were non-virulent to guinea pigs, were documented (Runyon, 1959). This observation along with the failure to find alternative etiological agents on bacteriologic and histopathologic investigations provided a strong clue that these strains produced disease in humans. In 1959 Ernst Runyon (Runyon, 1959) wrote “The guinea pig no longer sits alone on the throne of decision as to pathogenicity of acid-fast bacilli for man; there is the mouse, and also an empty chair. Mice have been found to be more susceptible than guinea pigs to some of the anonymous mycobacteria. The “empty chair” pertains to the lack of any known animal host in which certain strains can establish progressive infection, although these bacteria evidently have been involved in human disease” (Runyon, 1959).

**Table 1:** A historical account of discovery of nontuberculous mycobacteria and treatment of nontuberculous mycobacterial disease.

Year	Discovery	Reference
1885	Smegma bacillus	(Karlson and Feldman, 1953)
1900	Report of a rapidly growing AFB (probably *M.fortuitum*) from the tonsillar crypt and from the sputum of another patient	(Runyon, 1959)
1904	First report of a chronic injection site abscess caused by AFB	(Alvarez and Tavel, 1885)
1918	AFB were noted in the chronic pustular skin lesions of an English soldier who had been wounded in battle and then torpedoed in the North Sea	(Marzinowski, 1900)
1925	Early efforts at classification and differentiation of mycobacteria	(Ophüls, 1904)
1926	Description and naming of *M.marinum* as the cause of a disease in saltwater fish in the Philadelphia aquarium	(Aronson, 1926)
1933	Review of literature on NTM from human material	(Branch, 1933)
1938	Description and naming of *M.fortuitum*	(da Costa Cruz, 1938)
1943	An avian-like bacillus isolated from a man with silicotuberculosis was isolated. This organism was later shown to be *M.avium* serotype 2	(Runyon, 1959)
1948	Description of a new mycobacterial disease of man caused by bacillus now known as *M.ulcerans*	(Cobbett, 1918)
1951	First reporting the AFB now known as *M.intracellular* and a case of disseminated disease attributed to it, and the second the rediscovery of *M.marinum* (under a new name) as a cause of superficial skin lesions in man	(Wilson, 1925; MacCallum et al., 1948)
1952-1953	An AFB of the M.*fortuitum* complex, called *M.abscessus* was considered to be the cause of arthritis of the knee and superficial abscesses in a patient described in 1953. Pulmonary disease associated with achalasia of the cardia and described the *M. fortuitum-*like organisms that parasitized the damaged lung.	(Cuttino and McCabe, 1949; Norden and Linell, 1951; Tarshis and Frisch, 1952a; Tarshis and Frisch, 1952b; Tarshis and Frisch, 1952c; Moore and Frerichs, 1953)
1953	An account of 2 cases of “yellow bacillus” disease and the descriptions of the organism now called *Mycobacterium kansasii*	(Gibson, 1953)
1953	Non-specific nature of the weak skin reactions to tuberculin PPD seen in some populations might be due to infection with mycobacteria other than mammalian tubercle bacilli	(Lominski and Harper, 1953)
1954	Correlation of the known facts about the relationship between human pulmonary disease, NTM, and provided the first working classification of the organisms	(Buhler and Pollak, 1953)
1959	Runyon originally proposed that the strains virulent for chickens and rabbits be called *M. avium*, and those avium-like strains without virulence be called “Battey bacilli” (derived from the Battey State Hospital in Georgia)	(Ravindran et al., 2025)
1990	Official Statement of the ATS 1990	(Palmer, 1953)
1997	Official Statement (revised) of the ATS 1997	(Timpe and Runyon, 1954)
1999	Joint Tuberculosis Committee of the BTS guidelines 1999	(Anonymous, 1990)
2007	Official Statement of the ATS 2007	(Anonymous, 1997)
2016	The US Cystic Fibrosis Foundation and European Cystic Fibrosis Society guidelines consensus recommendations for the management of NTM in individuals with cystic fibrosis	(Hegde et al., 2024)
2017	BTS guidelines 2017	(Subcommittee of the Joint Tuberculosis Committee of the British Thoracic Society. Management of opportunist mycobacterial infections: Joint Tuberculosis Committee Guidelines 1999, 2000)
2020	ATS/ERS/ESCMID/IDSA guidelines	(Griffith et al., 2007)
2022	Consensus management recommendations for less common non-tuberculous mycobacterial pulmonary diseases	(Haworth et al., 2017)

AFB, acid-fast bacilli; NTM, nontuberculous mycobacterial disease; PPD, purified protein derivative; ATS, American Thoracic Society; ERS, European Respiratory Society; ESCMID = European Society of Clinical Microbiology and Infectious Diseases; IDSA, Infectious Diseases Society of America.

Thorough preparations for this survey were made. Particularly, the efforts by William H. Feldman circulated outlines for the study of these bacteria in 1953 are noteworthy. The Veterans Administration (VA) - National Tuberculosis Association (NTA) Survey of Mycobacterial Infections initiated in 1954 by a subcommittee of the VA Committee on Bacteriology with W.E. Dye as Chairman provided a great impetus to the understanding of the clinical significance of various mycobacteria (Timpe and Runyon, 1954; Runyon, 1955; Runyon, 1959). Ernst Runyon, the Chairman of VA subcommittee, had obtained the collaboration of the American Trudeau Society of the NTA in the distribution of a questionnaire and request for cultures (Runyon, 1955). In this survey (Runyon, 1955) of the assembled cultures, established Groups I (photochromogens), II (scotochromogens), III (nonphotochromogens) and IV (rapid growers) as useful features for differentiating the anonymous strains from *M. tuberculosis.* Aforementioned grouping of NTM was used for a long time and now is of historical significance. This grouping has been replaced by molecular methods for speciation and sub-speciation. In light of this survey, it became evident that clinical, radiographic and histopathologic studies are insufficient to differentiate pulmonary diseases due to various distinct types of anonymous (“atypical”) acid-fast organisms from each other and from TB; bacteriologic identification is essential. NTM from Groups I and III were noted to be the most important pathogens; geographical variations were evident. In the 1960s, studies from Mexico (Bojalil et al., 1962), USA (Wayne, 1966), and Japan (Tsukamura, 1967) reported new phenotypic traits allowing the identification of novel mycobacterial species, as well as improved understanding of epidemiology and pathogenicity of these organisms.

Wolinsky (1981) had addressed the issue of colonization *versus* infection in the context of these atypical organisms. He stated that while *M. tuberculosis*, a definitive pathogen, is rarely recovered from culture except in association with disease; NTM that are plentiful in the environment are often encountered as colonizers, or agents producing infection but not recognizable disease. Further, their propensity to cause opportunistic infection in previously damaged lung tissue makes it difficult to distinguish new disease from old. Wolinsky (1981) stated that the decision whether to treat disease caused by these atypical organisms or not rests with the physician and mentions following helpful facts to decide treatment. These include (i) moderate to heavy growth of the mycobacteria from sputum specimens (light growth may occur from sterile body fluids or tissues); (ii) repeated isolation of the same organism (in pulmonary disease); (iii) site of origin of the positive specimen; (iv) species/subspecies of mycobacteria identified; and (v) host risk factors/predisposing conditions. Wolinsky (1981) suggested that even when disease is determined it may be prudent to withhold treatment unless the disease progresses rapidly. This is still relevant presently as after isolation a greater emphasis is given to clinical relevance of isolated NTM species/subspecies (Daley et al., 2020).

In a 1979, in a state-of-the-art review (Wolinsky, 1979) titled, ‘Nontuberculous mycobacteria and associated diseases’ in the American Review of Respiratory Disease (an official publication of the American Thoracic Society [ATS], now named American Journal of Respiratory and Critical Care Medicine) Emanuel Wolinsky changed the perception about NTM and their potential to produce human diseases. By this time, 40 NTM species had been identified with the help of phenotypic and biochemical characteristics. This review article (Wolinsky, 1979) described virulence potential of these mycobacteria, and since then, this topic had been explored extensively by several researchers across the globe with a phenomenal momentum. The incidence of disseminated MAC rose dramatically in the pre-cART era, particularly in patients with CD4+ T-lymphocyte counts <50 cells/mm³ but declined substantially following the introduction of effective combination antiretroviral therapy and routine primary prophylaxis. In high-income settings, disseminated MAC is now concentrated in patients with advanced, untreated HIV infection and in those with poor treatment adherence. In contrast, pulmonary NTM disease has emerged as a major problem in older, structurally abnormal lungs, even in HIV-negative populations. Consistent with these trends, current guidelines recommend macrolide or rifabutin prophylaxis for patients with advanced immunosuppression (e.g. per NACO) alongside early ART and careful evaluation of any mycobacterial isolate to distinguish disseminated MAC from TB (Panel on opportunistic infections in adults and adolescents with HIV. Guidelines for the prevention and treatment of opportunistic infections in adults and adolescents with HIV: recommendations from the centers for disease control and prevention, the national institutes of health, and the HIV medicine association of the infectious diseases society of America). In the late 1980s, the International Working Group on Mycobacterial Taxonomy (IWGMT) coined the term “nontuberculous mycobacteria (NTM)” which has been widely accepted and used to date by various microbiologists and clinicians worldwide (Wayne, 1988). Several researchers are working either singly or in collaboration mode, nationally or at the international level. The speed and accuracy of mycobacterial species identification showed a significant improvement with the application of high-performance liquid chromatography (HPLC) for documenting the chromatographic profile of mycolic acid extracted from the bacterial cell wall and subsequent introduction of various molecular methods such as DNA probes and gene sequencing. After the advent of whole genome sequencing in 1987 the International Committee for Systematic Bacteriology defined that a species includes strains with ~70% or more deoxyribonucleic acid (DNA)-DNA homology and with 5 °C or less difference in melting temperature of the homologous genomic DNA and hybrid DNA (Δ*Tm)* providing the precise difference between genetically closed NTM species (Wayne, 1988). Conventionally NTM are divided into slowly growing mycobacteria (SGM; ≥7 days) and rapidly growing mycobacteria (RGM; <7 days) (Runyon, 1959). In molecular taxonomy, this has been attributed to the differences of 2–4 nucleotides in helix 18 of the hypervariable region B of the 16S ribosomal ribonucleic acid (rRNA) gene (Pereira et al., 2020). With the availability of gene sequencing for NTM speciation and a wide range of databases of mycobacterial species, in the last three decades the number of NTM species expanded more than three times (Turenne, 2019). Although nearly 200 NTM species have been described, only a few of these are pathogenic to causes diseases in humans (Turenne, 2019).

Friedmann’s discovery of *M. chelonae* (“chelonae”, Latin for “of a turtle”) in the lungs of two sea turtles in the early 20^th^ century marked the beginning of the history of the major pathogenic species of rapidly growing mycobacteria (RGM) (Cobbett, 1918; Brown-Elliott and Philley, 2017). The closely related *M. abscessus* was initially identified as the cause of a human skin and soft tissue infection in a patient who had several lower extremities soft tissue abscesses nearly 50 years later (Moore and Frerichs, 1953). Another RGM species, *M. fortuitum* (formerly *M. ranae*), was originally recovered from frogs in 1905. However, in 1938, da Costa Cruz (1938) named *M. fortuitum* to an isolate considering it a new mycobacterial species isolated from a patient with a skin abscess from local vitamin injections site. Subsequently, both organisms were proven to be the same, with the illegitimate name *M. fortuitum* retained as the species name. Early taxonomic studies based on phenotypic analysis concluded that the two species *M. fortuitum* and *M. chelonae* were composed of several “subspecies” (i.e., *M. chelonae* subsp. *chelonae* and *M. chelonae* subsp. abscessus) or biovars (*M. fortuitum* bv. *fortuitum*, *M. fortuitum* bv. *peregrinum*, and *M. fortuitum* third biovariant complex). In early 1980s, DNA-DNA hybridisation and 16S rRNA gene analysis revealed that *M. chelonae* and *M. fortuitum* biovars and subspecies are in fact different species. Until 1992, *M. abscessus* was considered as a subspecies of *M. chelonae*, however, after extensive genomic analysis in previous decade, *M. chelonae and M. abscessus* were designated as a different species (Kusunoki and Ezaki, 1992).

In view of increasing isolation rates and the establishment of various NTM as pathogens for humans, the first Official Statement of the ATS for diagnosis and treatment of NTM was released in 1990 (Anonymous, 1990) which was further revised in 1997 (Anonymous, 1997). The Joint Tuberculosis Committee of the British Thoracic Society (BTS) came up with an evidence-based document namely ‘Management of opportunist mycobacterial infections: Joint Tuberculosis Committee guidelines 1999’, which was published in the year 2000 (Subcommittee of the Joint Tuberculosis Committee of the British Thoracic Society. Management of opportunist mycobacterial infections: Joint Tuberculosis Committee Guidelines 1999, 2000). In 2007, the ATS issued for the first time landmark guidelines that addressed the diagnosis, treatment, and prevention of both NTM pulmonary disease (NTM-PD) and extra-pulmonary NTM disease (EP-NTM) (Griffith et al., 2007). Thereafter, in 2016, the consensus recommendations for the screening, investigation, diagnosis and management of NTM-PD in individuals with CF were issued by the US Cystic Fibrosis Foundation and European Cystic Fibrosis Society (ECFS) (Floto et al., 2016). Following that, two new guidelines, BTS 2017 (Haworth et al., 2017) and the ATS/European Respiratory Society (ERS)/European Society of Clinical Microbiology and Infectious Diseases (ESCMID)/Infectious Diseases Society of America (IDSA) 2020 (Daley et al., 2020), were released. The last two guidelines (Haworth et al., 2017; Daley et al., 2020) have exclusively focused on NTM-PD in people without HIV/AIDS or cystic fibrosis. Both guidelines endorsed the clinical, radiographic and microbiological criteria defined in the ATS (2007) guideline (Griffith et al., 2007) for diagnosing NTM-PD. Subsequently, the panel members of the 2020 ATS, ERS, ESCMID, and IDSA Guideline Committee also released ‘Consensus management recommendations for less common NTM-PD’ in 2022 (Lange et al., 2022).

## 3 Nontuberculous mycobacterial diseases in India

In India, there has been no coherent historical time-line of NTM prevalence due to a lack of surveillance data and a near-total absence of research mapping environmental reservoirs in water or soil. A significant policy gap meant NTM remained invisible to the public health system. Unlike TB, India has historically had no national program or country-specific guidelines for NTM diagnosis and treatment, forcing clinicians to rely on international standards that may be ill-suited for the local context of drug availability and cost. The historical interaction between human, animal, and environmental NTM remains completely unexplored. The “One Health” perspective—linking human, animal, and environmental health—is a recent development (Hegde et al., 2024) The historical interaction and potential transmission between animals and humans in the Indian context is completely unknown.

**Figure 3:**
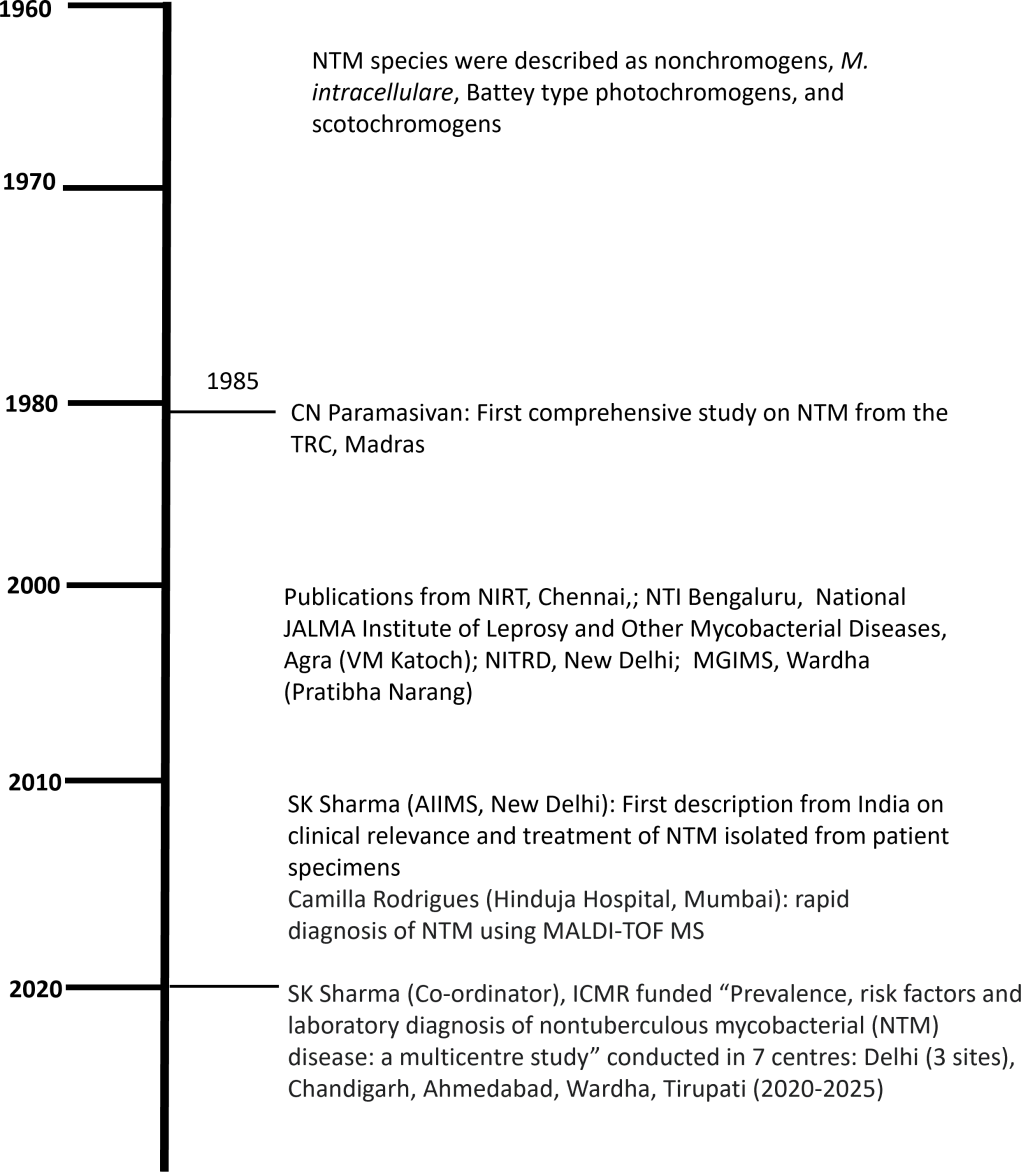
History of nontuberculous mycobacteria: India. TRC, Madras, Tuberculosis Research Centre (now called National Institute for Research in Tuberculosis, Chennai); NTI, National Tuberculosis Institute; NITRD, National Institute of Tuberculosis and Respiratory Diseases; AIIMS, New Delhi, All India Institute of Medical Sciences; MGIMS, Mahatma Gandhi Institute of Medical Sciences, Wardha (Maharashtra); MALDI TOF MS, matrix assisted laser desorption time of flight mass spectrometry; ICMR, Indian Council of Medical Research.

**Figure 4:**
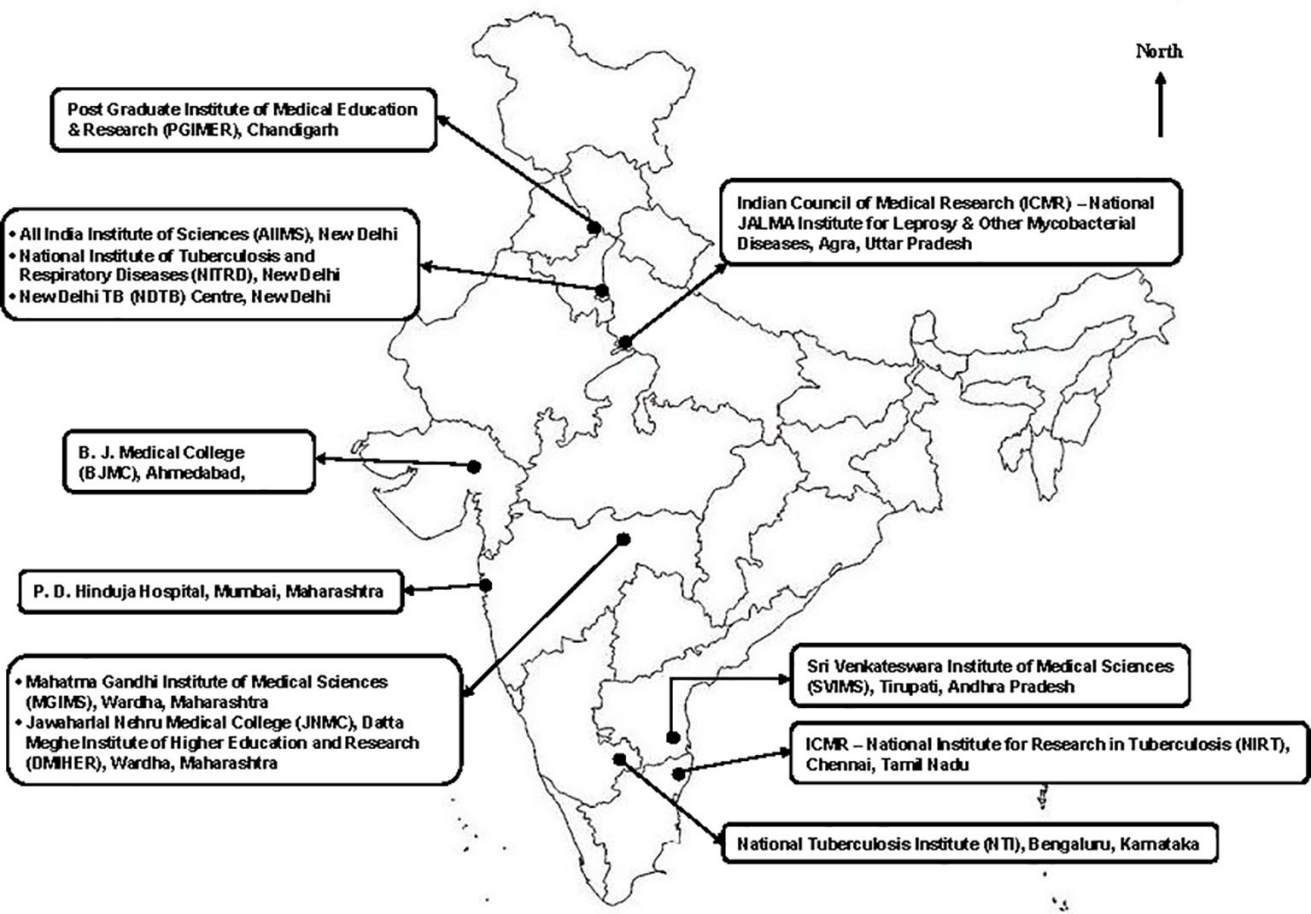
Locations of major Indian laboratories and hospitals reporting NTM studies.

A few studies (Narain et al., 1968; Rao and Kotian, 1976; Mustafa and Talwar, 1978; Choudhri et al., 1979; Kotian et al., 1981; Ramakrishnan, 1981) on NTM were published between 1961 and 1981 from India in which most NTM species were described as nonchromogens, *M. intracellulare*, Battey type photochromogens, and scotochromogens. A timeline of key landmarks from India in the field of NTM is provided in **Figure 3**. In 1981, C.N. Paramasivan, a medical microbiologist and pioneer in the field of NTM research in India, published the first comprehensive study from the Tuberculosis Research Centre, Madras (now known as National Institute for Research in Tuberculosis [NIRT], Chennai) (Paramasivan et al., 1985). Though occasional original articles/research articles on NTM had been published thereafter (Shanker et al., 1989; Karak et al., 1996), work on NTM in India peaked in the 2000s. Work reported from several national TB diagnostic laboratories (**Figure 4**) (Jain et al., 1991; Gupta et al., 2002; Narang et al., 2009; Parashar et al., 2009; Garima et al., 2012; Mishra et al., 2018; Sebastian et al., 2018a; Sebastian et al., 2018b; Sharma and Upadhyay, 2020; Shrivastava et al., 2020) contributed to spreading awareness about the various methods of NTM isolation and speciation among microbiologists in India. In that era, biochemical assays that were cumbersome and time-consuming had been used for speciation and sub-speciation. However, most of the data reported from these centers had emanated from laboratories, often based on a single time isolation, without establishing the clinical relevance of NTM isolates (Wolinsky, 1981; Narang et al., 2009; Mishra et al., 2018; Sharma and Upadhyay, 2020). No serious efforts were made to record and report their clinical details, or to trace these patients for their treatment. Though some studies (Jesudason and Gladstone, 2005; Maurya et al., 2015; Radha Bai Prabhu et al., 2019; Arvind et al., 2020; Gupta et al., 2020; Singh et al., 2020; Wani et al., 2020; Sali et al., 2021; Thangavelu et al., 2021; Das et al., 2022; Dadheech et al., 2023) were published subsequently, these have not systematically documented clinical and radiographic involvement in NTM patients.

Researchers from the All India Institute of Medical Sciences (AIIMS), New Delhi (Singh et al., 2007; Khatter et al., 2008; Jain et al., 2014; Sharma et al., 2019; Wazahat et al., 2023) had contributed to the understanding of NTM disease from India. During the last decade, the research group led by Sharma (Sharma et al., 2019) was the first to publish data from India describing the “clinical relevance and treatment outcomes” of NTM isolated from patient specimens at AIIMS, New Delhi (Sharma et al., 2019). “Pseudo outbreaks” of health-care associated NTM-PD due to use of tap water to clean wounds, instruments is common; tap water is the major reservoir for MAC, *M. kansasii, M. gordonae, M. xenopi, M. abscessus, M. fortuitum, M. chelonae, M. scrofulaceum, M. kansasii* (Satta Y, et al., 2020; Sharma and Upadhyay, 2020). They had also advocated the role of molecular diagnosis of NTM using line probe assay and targeted gene sequencing (Sharma et al., 2019). The research group led by Camilla Rodrigues, Mumbai had also contributed to NTM research and worked on rapid diagnosis of NTM using matrix-assisted laser desorption/ionization-time of flight mass spectrophotometry (MALDI-TOF MS) (Shenai et al., 2009; Shenai et al., 2010; Jani et al., 2011; Rodrigues, 2015). There have also been occasional publications from the north-eastern states of India, namely, Arunachal Pradesh (Mudliar et al., 2022), Assam (Kalita et al., 2005), Meghalaya (Vise et al., 2016), Mizoram (De Mandal et al., 2015), and Tripura (Bhattacharya et al., 2021).

The first-of-its-kind, multicenter research project (Sharma et al., 2025) of national importance on NTM disease funded by the Indian Council of Medical Research (ICMR), Ministry of Health & Family Welfare, Government of India revealed that NTM were isolated from 226/71,143 (0.32%) TB suspects. In NTM-pulmonary disease (PD) (n=187), most common species isolated were *M. avium complex* (MAC) (27.8%), followed by *M. kansasii* (24.1%), *M. abscessus* (21.4%); in EP-NTM disease (n=39), *M. abscessus* (41%), *M. fortuitum* (41%), and MAC (15.4%) were the most common species isolated. This study is expected to provide a detailed account of prevalence, risk factors, clinical presentation, imaging features, laboratory diagnosis, and treatment outcomes of NTM-PD and EP-NTM (Sharma et al., 2025).

The first case of HIV in India was reported from Chennai in 1987 (Simoes et al., 1987). Subsequently, recognizing the importance of the deadly interaction between TB and HIV, the National (policy) Framework for Joint TB/HIV Collaborative Activities was jointly developed by the Revised National TB Control Programme (RNTCP) (now called National TB Elimination Programme [NTEP]) and the National AIDS Control Programme (NACP), bidirectional screening for HIV and TB was initiated (Department of AIDS Control et al., [[NoYear]]). In high TB burden countries including India, where TB is much more common, it is often difficult to differentiate pulmonary TB from the NTM-PD. The picture gets further complicated when sputum smear microscopy was extensively used for the diagnosis and treatment of TB in National TB Control Programmes, because of non-availability of mycobacterial culture facilities in high TB burden countries. These sputum smear-positive patients were often started on anti-TB drugs on the basis of sputum smear microscopy results. In the absence of mycobacterial culture, it is possible that several of these sputum smear-positive patients treated for TB would in fact have been patients with NTM-PD that were missed. The important differences between TB and NTM disease are shown in **Supplementary Table 1** (Sharma and Upadhyay, 2020).

## 4 Immunopathogenesis of NTM disease

Immunopathogenesis of NTM disease is not completely understood because earlier literature lacked species and subspecies−level identification, surveillance, and environmental mapping, obscuring links between particular NTM, host factors, and characteristic immune phenotypes relative to tuberculosis (TB) (CruzAguilar et al., 2021; Lyu, 2024; Chancharoenthana et al., 2025). Modern liquid culture, probe−based and PCR diagnostics, and species−specific epidemiology revealed heterogeneity in virulence attributes such as biofilm formation, glycopeptidolipid (GPL) masking, and phagosome−level evasion, while cohort immunophenotyping uncovered variable Th1 tone, innate receptor signaling, and exhaustion signatures across clinical phenotypes (CruzAguilar et al., 2021; Lyu, 2024; Chancharoenthana et al., 2025). These advances explain why unified models are elusive and why species, morphotype, host structure, and immunogenetics must be integrated to describe NTM pathogenesis, in contrast to the more stereotyped, necrotizing Th1 pathology of human−adapted *M. tuberculosis* (**Table 2**) (Wu and Holland, 2015; Lyu, 2024; Chancharoenthana et al., 2025). Key immunopathogenic contrasts between nontuberculous mycobacteria and *M. tuberculosis* are summarized in **Table 2**.

**Table 2:** Differences between immunopathogenesis of NTM and TB disease.

Domain	Nontuberculous mycobacteria	*Mycobacterium tuberculosis*
Reservoir and transmission	Environmental water/soil/biofilms with exposure-driven acquisition; person-to-person spread is not dominant	Human reservoir with predominant airborne transmission and high adaptation to human hosts
Typical host risk profile	Structural lung disease, impaired airway clearance, elderly women with thoracic dysmorphisms, and cellular immune defects, including anti–IFN-γ autoantibodies and Mendelian susceptibility to mycobacterial disease variants	Severe disease is frequent in immunocompetent hosts; risk amplified by human immunodeficiency virus and iatrogenic immunosuppression
Innate recognition and activation	PRR signaling (TLR2-centered) with heterogeneous IL-12–IFN-γ coupling and variable macrophage activation by species and host	Robust PRR signaling and IL-12–IFN-γ coupling drive strong Th1 responses
Intracellular fate	MAC blocks phagosome-lysosome fusion; *M. abscessus* rough damages phagosomes and spreads; rapid growers emphasize extracellular biofilms	Phagosomal arrest with canonical survival programs promotes intracellular persistence and necrotizing pathology
Granuloma morphology	Often chronic, fibrotic, and less caseating with prolonged host–pathogen equilibrium	Frequently, caseating granulomas with cavitation and high bacillary burden
Immune evasion	GPL masking of TLR ligands, phagolysosome blockade, cytokine modulation, phagosomal damage/escape, and biofilm-mediated tolerance, varying by species/morphotype	Potent inhibition of phagosome maturation and manipulation of host inflammation and cell death
Autophagy/xenophagy	Frequent flux impairment and non-canonical routing; TFEB activation (amiodarone, rufomycins, resveratrol analog V46), trehalose–PIKFYVE–TFEB, and AMPK activation (metformin) restrict intracellular bacilli; macrolides can alkalinize lysosomes and oppose killing in *M. abscessus*	Xenophagy is engaged, but acidification and degradation curtailed; vitamin D–CAMP, autophagy initiation, and TFEB activation reduce burden in models
Microbiome influence	Gut–lung axis and airway microbiota modulate immune tone and treatment responsiveness in NTM-PD	Microbiome effects are reported but less central to classical transmissibility and caseating pathology paradigms
Extrapulmonary pathogenesis	Direct inoculation and medical instrument-associated biofilms drive skin/soft-tissue, lymphatic, and musculoskeletal disease; hematogenous dissemination in IL-12–IFN-γ axis defects or anti–IFN-γ autoantibodies produces multi-organ disease with high organism load	Hematogenous and lymphatic spread frequently produce caseating granulomas in lymph nodes, bones, and viscera with strong necrotizing Th1 pathology, even without device biofilms

NTM, nontuberculous mycobacteria; TB, tuberculosis; PRR, pattern recognition receptor; TLR2, Toll−like receptor 2; PAMP, pathogen−associated molecular pattern; IL−12, interleukin−12; IFN−γ, interferon−gamma; Th1, type 1 helper T cell; MAC, *Mycobacterium avium* complex; GPL, glycopeptidolipid; RGM, rapidly growing mycobacteria; LC3, microtubule−associated protein 1 light chain 3; p62, sequestosome−1; OPTN, optineurin; NBR1, neighbor of BRCA1 gene 1; NDP52, nuclear dot protein 52 kDa; TFEB, transcription factor EB; AMPK, AMP−activated protein kinase; PIKFYVE, phosphatidylinositol−3−phosphate 5−kinase; CAMP, cathelicidin antimicrobial peptide; ESX−1, ESAT−6 secretion system−1; ESCRT, Endosomal Sorting Complex Required for Transport; LAMP1, lysosome−associated membrane protein 1; ROS, reactive oxygen species; RNS, reactive nitrogen species; HIV, human immunodeficiency virus; SSTI, skin and soft−tissue infection; NTM−PD, nontuberculous mycobacterial pulmonary disease.

*Innate and adaptive immunity*

Inhaled NTM adhere to mucus and compromised epithelium and are phagocytosed by alveolar macrophages that recognize PAMPs via PRRs, prominently TLR2, initiating IL−12–IFN−γ signaling, TNF production, and chemokine programs that recruit neutrophils, monocytes, NK cells, and lymphocytes to nascent lesions (Lyu, 2024; Chancharoenthana et al., 2025). In many patients with NTM−D, TLR2 expression and downstream IL−12 and TNF production are diminished in response to MAC stimulation, reflecting subtle innate deficits that bias toward persistence rather than sterilization at the macrophage–granuloma interface (CruzAguilar et al., 2021; Lyu, 2024; Chancharoenthana et al., 2025).

In contrast to TB’s frequent caseating necrosis, NTM granulomas show chronic inflammation and fibrosis with comparatively less caseation, consistent with heterogeneous Th1 drive, prolonged host–pathogen equilibrium in structurally abnormal lungs, and species−specific blockade of phagolysosomal killing (**Table 3**) (Lyu, 2024; Chancharoenthana et al., 2025). Adaptive control hinges on Th1 polarization driven by IL−12–IFN−γ that activates macrophage microbicidal programs and sustains organized granulomas, while Th17 pathways aid mucosal defense and neutrophil recruitment but can also amplify airway injury in bronchiectasis and cystic fibrosis when dysregulated (Lyu, 2024; Chancharoenthana et al., 2025). Disseminated NTM often unmasks inborn or acquired defects along the IL−12–IFN−γ axis, and even in pulmonary disease without overt inborn errors, signatures of T−cell exhaustion and altered cytokines point to broader immune dysregulation underlying chronic infection trajectories. Clinically, MAC typically impedes phagosome–lysosome fusion and neutralizes antimicrobial effectors to persist within vacuoles, whereas *M. abscessus* smooth variants use GPLs to dampen TLR2 sensing and rough (more virulent) variants damage phagosomes, induce type I IFN, and spread cell−to−cell, mapping distinct evasion modes to nodular−bronchiectatic versus rapidly destructive phenotypes.

**Table 3:** Comprehensive timeline and developmental status of antimicrobial agents used or investigated for nontuberculous mycobacterial (NTM) infections.

Drug (Year of discovery)/ Route of administration	Mechanism of action	Year of first use		Remarks in the context of NTM	Current status in NTM treatment	Reference for first use in NTM diseases
		TB or other infections	NTM			
Streptomycin (1943)/ Parenteral	Protein synthesis inhibitor (30S ribosomal subunit)	1943 (TB)	1947	First antibiotic for mycobacterial diseases	Historically approved	(Simoes et al., 1987)
PAS (1902)/ Oral	Folate synthesis inhibitor	1943 (TB)	1957	Early antimycobacterial, repurposed from TB; limited current use	Historically approved	(Department of AIDS Control et al., 2013)
Isoniazid (1912)/ Oral	Inhibits mycolic acid synthesis	1943 (TB)	1957	Widely tried, effective for a few NTM species	Historically approved	(CruzAguilar et al., 2021)
Clofazimine (1954)/ Oral	Disrupts bacterial respiration & ion transport	1962 (Leprosy)	1964	Adjunct in refractory NTM	Standard-of-care	(Chancharoenthana et al., 2025)
Ethionamide (1956)/ Oral	Inhibits mycolic acid synthesis	1965 (TB)	1965	Occasionally used in resistant cases	Historically approved	(Lyu, 2024)
Rifampicin (1957)/ Oral	Inhibits DNA-dependent RNA polymerase	1967 (TB)	1969	Backbone of MAC therapy	Standard-of-care	(Kim et al., 2025)
Ethambutol (1961)/ Oral	Inhibits arabinosyl transferase	1969 (TB)	1969	Used in MAC combinations	Standard-of-care	(Thompson et al., 2025)
Doxycycline (1958)/ Oral and parenteral	Protein synthesis inhibitor (30S ribosomal subunit)	1958 (Bacterial infections)	1981	Used off-label for rapidly growing mycobacteria, particularly *M. fortuitum* and *M. chelonae*; typically, in combination regimens; limited efficacy for *M. abscessus*	Standard-of-care (for select rapidly growing species)	(Wu and Holland, 2015)
Amikacin (1972)/ Parenteral	Protein synthesis inhibitor (30S ribosomal subunit)	1981 (TB)	1981	For severe and refractory NTM pulmonary disease.	Standard-of-care	(Wu and Holland, 2015)
Cefoxitin (1972)/ Parenteral	Cell wall synthesis inhibitor (β-lactam)	1974 (Bacterial infections)	1990	Used in combination therapy for *M. abscessus*; off-label use	Standard-of-care	(Clinical and Laboratory Standards Institute, 2018)
Ciprofloxacin (1983)/ Oral and parenteral	DNA gyrase inhibitor	1986 (Broad-spectrum infections)	1986	Early fluoroquinolone for NTM; off-label use	Standard-of-care	(Maclean and Smith, 1947)
Rifabutin (1975)/ Oral	Inhibits DNA-dependent RNA polymerase	1990 (TB)	1990	Alternative rifamycin for MAC & *M. abscessus*;	Standard-of-care	(Wolinsky et al., 1957)
Clarithromycin (1980)/ Oral and parenteral	Protein synthesis inhibitor (50S ribosomal subunit)	1990 (Respiratory infections)	1990	First effective macrolide for MAC/NTM	Standard-of-care	(Crow et al., 1957)
Azithromycin (1980)/ Oral and parenteral	Protein synthesis inhibitor (50S ribosomal subunit)	1992 (Respiratory infections)	1992	Preferred macrolide for NTM treatment; better tolerance	Standard-of-care	(Lunn and Rees, 1964)
Imipenem (1985)/ Parenteral	Cell wall synthesis inhibitor (β-lactam)	1995 (Bacterial infections)	1995	Rescue therapy in drug-resistant NTM; off-label use	Standard-of-care	(Carruthers and Edwards, 1965)
Levofloxacin (1991)/ Oral and parenteral	DNA gyrase inhibitor	1996 (Broad-spectrum infections)	1996	Alternative fluoroquinolone for NTM; off-label use	Standard-of-care	(Jones, 1969)
Linezolid (1996)/ Oral/Parenteral	Protein synthesis inhibitor (50S ribosomal subunit)	2001 (Gram-positive infections)	2001	Salvage therapy for refractory NTM; off-label use	Standard-of-care	(Rothstein, 1969)
Moxifloxacin (1996)/ Oral and parenteral	DNA gyrase inhibitor	2003 (Broad-spectrum infections)	2003	Improved fluoroquinolone for select NTM; off-label use	Standard-of-care	(Dalovisio et al., 1981)
Bedaquiline (2005)/ Oral	Inhibitor of ATP synthase	2006 (TB)	2012	Experimental for NTM, especially for *M. abscessus*	Phase 2 and 3 trial	(Becker et al., 1980)
Tigecycline (1999)/ Parenteral	Protein synthesis inhibitor (30S ribosomal subunit)	2005 (Broad-spectrum infections)	2007	Salvage therapy for multidrug-resistant NTM; off-label	Standard-of-care	(Young et al., 1986)
Tedizolid (2005)/ Oral	Protein synthesis inhibitor (50S ribosomal subunit)	2017 (Gram-positive infections)	2017	Less toxic alternative to linezolid; off-label use	Standard-of-care	(Snider et al., 1987)
GM-CSF (1980s)/ Inhalational	Immunomodulator; enhances alveolar macrophage activation	2018 (Immunotherapy)	2018	Off-label, adjuvant immunotherapy in refractory NTM	Approved – Phase IV trial(off-label)	(Bessesen et al., 1990)
Amikacin liposome inhalation suspension (ALIS) (2018)/ Inhalational	Protein synthesis inhibitor (30S ribosomal subunit)	2018 (NTM)	2018	Approved inhaled formulation for refractory MAC	Approved, Phase III/IV trial	(Young et al., 1991)
Bacteriophage therapy (1920s)/ Parenteral and topical	Lytic bacteriophages targeting mycobacteria	2019 (Bacterial infections)	2019	Experimental; compassionate use in MDR NTM	Investigational, compassionate use/case reports	(Woods and Washington, 1987)
Inhaled clofazimine (1954)/ Inhalational	Disrupts bacterial respiration & ion transport	2020 (Inhalation)	2020	Repurposed formulation for lung targeting in NTM	Investigational – Phase 2 trial	(Rastogi et al., 1996)
Omadacycline (2014)/ Oral/Parenteral	Protein synthesis inhibitor (30S ribosomal subunit)	(2021) Skin and respiratory infections	2021	Off-label; exploratory use against rapidly growing mycobacteria	Phase 2 trial	(Brown-Elliott et al., 2001)

NTM, nontuberculous mycobacteria; TB, tuberculosis; MAC, *M. avium* Complex; PAS, para-amino salicylic acid; IV, Intravenous; MDR, multidrug-resistant; ATP, adenosine triphosphate; GM-CSF, granulocyte-macrophage colony-stimulating factor; RGM, rapidly growing mycobacteria. The following drugs are not available in Indian markets as of current regulatory and clinical practice status: ALIS; bacteriophage therapy; inhaled clofazimine; inhaled GM-CSF; and omadacycline. Cefoxitin; ALIS; omadacycline; inhaled GM-CSF; inhaled clofazimine; and bacteriophage therapy are approved or used exclusively in the USA (not approved in India).

### 4.1 Autophagy and xenophagy in NTM infection

Macroautophagy and xenophagy tag intracellular bacilli with ubiquitin and recruit cargo receptors (p62, OPTN, NBR1, NDP52) to LC3−decorated membranes for autophagosome formation and lysosomal degradation, yet NTM frequently impair fusion, acidification, or flux to persist in macrophages. In MAC, the dominant phenotype is vacuolar residence with blockade of phagosome–lysosome fusion and neutralization of ROS/RNS, whereas *M. abscessus* shows morphotype−dependent autophagy biology: smooth (GPL−rich) variants attenuate early PRR signaling while rough variants trigger abundant autophagosomes yet damage phagosomal membranes, induce apoptosis and type I IFN, and spread cell−to−cell. Additional diversity arises in *M. marinum*, where ubiquitinated bacilli can be routed to LAMP1−positive compartments via non−canonical routes and ESCRT−mediated vacuolar repair while ESX−1 upregulates autophagy genes but suppresses cargo degradation. These mechanistic bottlenecks create host−directed therapy opportunities: TFEB–lysosomal activators (amiodarone, rufomycins, resveratrol analog V46), AMPK activation (metformin), and trehalose–PIKFYVE–TFEB signaling restore degradative capacity and reduce intracellular MAC and *M. abscessus* in models, whereas macrolide−induced lysosomal alkalinization can impair autophagy−dependent killing of *M. abscessus*, emphasizing the need to align host−directed therapy with species and regimen context (**Table 2**).

In TB, xenophagy is also engaged but acidification and flux are curtailed by M. tuberculosis, and augmentation through vitamin D–CAMP, autophagy initiation, and TFEB activation restricts bacillary burden in experimental systems, highlighting convergences and divergences from NTM autophagy biology (**Table 3**).

### 4.2 Gut–lung axis and NTM disease

Human cohorts reveal reduced gut microbial diversity and taxa shifts in NTM−PD correlating with diminished TLR2−linked signaling, immune exhaustion markers, and early treatment responsiveness differences, while experimental restoration of commensals such as Prevotella copri augments TLR signaling and reduces pulmonary susceptibility.These data suggest that immunometabolic–microbiome interactions contribute to heterogeneous inflammatory phenotypes, reinfection risk, and outcome variability in NTM−PD beyond pathogen−intrinsic virulence and host structure alone.

### 4.3 Species mechanisms beyond MAC and M. abscessus complex

Beyond MAC and *M. abscessus*, *M. kansasii* in immunocompetent hosts often mimics TB with cavitation and stronger Th1−associated pathology, reflecting robust PRR signaling and intracellular persistence with more pronounced necrosis than MAC in some cohorts (Lyu, 2024; Chancharoenthana et al., 2025). *M. xenopi* favors vacuolar persistence with impaired phagolysosome fusion, producing indolent granulomatous inflammation and fibrosis in structurally abnormal lungs, and *M. malmoense* produces chronic fibrotic granulomas and low−grade inflammation analogous to MAC. Rapid growers such as *M. fortuitum* and *M. chelonae* form biofilms on airway surfaces and devices, provoking neutrophil−predominant inflammation and extracellular tolerance where source control is essential alongside antimicrobials (Lyu, 2024; Chancharoenthana et al., 2025). *M. marinum* causes cutaneous granulomatous disease via ESX−1–mediated phagosomal damage and ESCRT−linked routing, while autophagic cargo degradation is suppressed *in vivo*. *M. ulcerans* secretes mycolactone, driving local immunosuppression through mTOR activation and autophagy suppression with extensive necrosis, immunologically distinct from NTM−PD. *M. haemophilum* and pediatric lymphadenitis species such as *M. scrofulaceum* show cooler temperature preference and iron dependence with nodal tropism or strong localized granulomatous responses emphasizing regionally constrained Th1 control.

### 4.4 Extrapulmonary NTM disease pathogenesis

Extrapulmonary disease follows two routes: direct inoculation from procedures/trauma/injections causing skin/soft−tissue infection and lymphadenitis, or hematogenous spread in hosts with compromised cell−mediated immunity, with device−associated biofilms sustaining chronic infection and necessitating source control plus prolonged, species−directed therapy (**Table 3**). Disseminated disease is classically associated with IL−12–IFN−γ axis defects and anti–IFN−γ autoantibodies, while musculoskeletal and prosthetic infections reflect extracellular biofilms and neutrophilic inflammation distinct from caseating granulomas of extrapulmonary TB (**Table 2**).

### 4.5 Disseminated NTM disease

Disseminated NTM is strongly linked to inborn errors in the IL−12–IFN−γ pathway, including autosomal mutations in IL12B, IL12RB1, ISG15, IFNGR1, IFNGR2, STAT1, and IRF8, X−linked defects in IKBKG (NEMO) and CYBB (gp91phox), plus GATA2 deficiency and anti–IFN−γ neutralizing autoantibodies, spanning childhood to adult susceptibility across localized and disseminated syndromes.

## 5 Evolution of treatment

High TB burden countries have prioritized healthcare systems towards TB elimination because of the importance of person-to-person transmission in pulmonary TB, which forms ~80% of TB cases in immunocompetent adults, while NTM is not transmitted from person-to-person, except *M. abscessus* in cystic fibrosis cases, which is rare in high TB burden countries. Further, unlike in the natural history of TB, where TB infection (TBI, previously called latent TB infection) and TB disease have been described, latent NTM infection does not occur (Sharma and Upadhyay, 2020; Sharma and Upadhyay, 2021). The treatment of NTM-PD and EP-NTM varies according to the isolated species and, in some patients, subspecies, disease severity, underlying comorbidities. Unlike in TB, *in vitro* drug-susceptibility testing (DST), often does not predict *in vitro* clinical response and is, therefore, not considered essential for management of NTM diseases. DST should follow the Clinical and Laboratory Standards Institute (CLSI) guidelines (Clinical and Laboratory Standards Institute, 2018). Like TB, treatment of NTM disease involves administration of multiple drug combinations. Treatment regimens comprise various antimycobacterial drugs that need to be administered daily if the NTM-PD is severe for long periods of time. Most antimicrobial agents used for NTM diseases have been repurposed from TB or other bacterial infections. Over time, their roles have evolved based on emerging evidence, clinical experience, and drug resistance patterns, shaping the current therapeutic landscape (**Table 3**) (Maclean and Smith, 1947; Crow et al., 1957; Wolinsky et al., 1957; Lunn and Rees, 1964; Carruthers and Edwards, 1965; Jones, 1969; Rothstein, 1969; Becker et al., 1980; Dalovisio et al., 1981; Young et al., 1986; Snider et al., 1987; Woods and Washington, 1987; Bessesen et al., 1990; Young et al., 1991; Rastogi et al., 1996; Brown-Elliott et al., 2001; Griffith et al., 2003; Rolfe et al., 2007; Philley et al., 2015; Yuste et al., 2017; Griffith et al., 2018; Scott et al., 2018; Dedrick et al., 2019; Kim et al., 2020; Mingora et al., 2023). Because of the 200 NTM species/subspecies, it is not possible to carry out randomized controlled clinical trials. As a result of this, there are no evidence-based guidelines for the treatment of NTM diseases. In high TB burden countries like India, patients with NTM-PD have extensive unilateral or bilateral lung involvement due to past history of TB and, therefore, surgery is not possible. In EP-NTM, surgical debridement and multidrug treatment are possible (Sharma et al., 2021).

Since the emergence of the 1990 ATS guidelines (Anonymous, 1990), guideline-based treatment (GBT) often involved a multi-drug regimen to be continued for 12 months even after culture conversion, whereas watchful waiting is recommended in specific cases where clinical relevance of isolated NTM is not clear. In 2017, a phase 2 clinical trial (Olivier et al., 2017) had shown that addition of amikacin liposome inhalation suspension (ALIS) that delivers high concentrations to the site of infection, limiting systemic exposure was found to increase culture conversion rates in patients with refractory NTM lung disease compared to GBT alone. Following encouraging results noted in this trial (Mingora et al., 2023) and another phase 3 trial published in 2018 (Griffith et al., 2018), addition of ALIS to GBT in patients who fail to convert after 6 months of treatment has been included in the ATS/ERS/ESCMID/IDSA clinical practice guideline (Daley et al., 2020).

It became known that organism is naturally resistant to pyrazinamide (a prodrug) due to reduced pyrazinamidase activity, preventing conversion of the drug into pyrazinoic acid (active bactericidal compound) (Sun and Zhang, 1999), pyrazinamide is not recommended for *M. kansasii* pulmonary disease. The total duration of treatment of *M. kansasii* and *M. szulgai* disease is 12 months, inclusive of culture conversion time period. In all other NTM species, treatment duration is for 12 months after culture conversion. The importance of an active erythromycin ribosomal methylase [*erm*] 41 gene in rapidly growing mycobacteria like *M. abscessus* in inducible resistance to macrolides was recognized in 1996 (Meier et al., 1996) and this has led to checking for *erm* (Wolinsky, 1979) gene status in *M. abscessus* in the later ensuing guidelines (Wallace et al., 1996).

Consequently, NTM-PD is associated with a huge socioeconomic burden on the patient. As a result, multidisciplinary teams are believed to be most suited for helping patients, providing adjunctive therapy in addition to GBT as needed. However, studies have indicated that in general compliance with GBT during the therapy of NTM-PD is very low. Surgery constitutes a first-line treatment option in NTM-PD if the patient has localised disease, good nutritional and cardiopulmonary functional status. The treatment of EP-NTM involves combining antimycobacterial therapy with possible surgery to achieve the best outcome. Adjunctive surgery is described as a possibility for selected individuals who have failed antibiotic treatment due to drug-resistant NTM isolates and drug-induced toxicity. Similar to the diagnosis, there are currently no published guidelines for the treatment of EP-NTM. Recently dated June 14, 2024, the NTEP, in the website “Knowledge Base for the National TB Elimination Program – NTEP”, under “Treatment regimen for Non-Mycobacterium tuberculosis (NTM) (ID 1791)” (https://ntep.in/node/1792/CP-treatment-regimen-ntm) provided “suggested treatment regimen covering maximum Non-Mycobacterium Tuberculosis (NTM) mainly MAC”. Overall, a lack of awareness among healthcare workers, inadequate laboratory infrastructure, and non-existent programmatic management constitutes obstacles in ensuring rational scientific diagnosis and treatment of NTM disease.

## 6 Biomarkers associated with NTM disease risk, treatment response, and prognosis

Culture-independent markers may be biological components of NTM or the host immune response to the organism (**Table 4**) (Schaefer, 1965; Szulga et al., 1966; Birn et al., 1967; Marks et al., 1969; Jenkins et al., 1971; Yoder and Schaefer, 1971; Jenkins and Marks, 1973; Brennan et al., 1978; Torrelles et al., 2002; Schorey and Sweet, 2008; Watanabe et al., 2011; Kitada et al., 2017; Han et al., 2020; Matsuda et al., 2020; Nithichanon et al., 2020; Steffen et al., 2020; Kawasaki et al., 2022; Mani-Varnosfaderani et al., 2022; Manbenmad et al., 2023; Park et al., 2023; Anh et al., 2024; Choi et al., 2024; Jia et al., 2024). To present, the most promising diagnostic and disease monitoring biomarkers include serum anti-glycopeptidolipid (GPL)-core antibodies [immunoglobulin A (IgA) and immunoglobulin G (IgG)]. Schaefer, Marks, and Jenkins’ work in the 1960s and 1970s laid the groundwork for identifying and characterizing GPLs (Rothstein, 1969) Later, it was demonstrated that thin-layer chromatography could be used to describe various membrane lipids of NTM and supplement the use of seroagglutination in NTM categorization (Schaefer, 1965; Szulga et al., 1966; Birn et al., 1967; Marks et al., 1969; Jenkins et al., 1971; Yoder and Schaefer, 1971; Jenkins and Marks, 1973). Patrick Brennan, a renowned microbiologist from the USA, and his colleagues discovered the mycobacterial glycopeptidolipid (GPL) structure in 1978–79 and continued to study its various aspects in different NTM species until early 2000 (Brennan et al., 1978; Torrelles et al., 2002). The GPLs are a class of glycolipids present on the outermost surfaces of several NTM, including MAC, *M. smegmatis*, *M. abscessus*, and *M. fortuitum*. GPL core structure consists of a 3-hydroxy or 3-methoxy C26-C33 fatty acyl chain N connected to a tripeptide-amino-alcohol composed of D-phenylalanine-D-allo-threonine-D-alanine-L-alaninol (Schorey and Sweet, 2008). The lipopeptide core is glycosylated with 6-deoxytalose (linked to allo-threonine) and α-L-rhamnose (linked to L-alaninol) (Schorey and Sweet, 2008). The resultant oligosaccharide residues are methylated to generate non-serovar-specific (apolar) GPLs (nsGPLs), which are present in all GPL-producing mycobacteria (Schorey and Sweet, 2008). However, MAC species also synthesize polar GPLs, in which some oligosaccharides residues are attached to the 6-deoxytalose-producing serovar-specific GPLs (ssGPLs) (Schorey and Sweet, 2008). Some RGM (*M. smegmatis, M. abscessus*, *M. fortuitum, and M. chelonae*) species also produce polar GPLs different from MAC due to the presence of 3,4-di-O-methyl rhamnose attached to the alaninol-linked 3,4-di-O-methyl rhamnose. By, contrast, these GPLs are not present in *Mtb* or *M. kansasii* (Schorey and Sweet, 2008). The levels of serum anti-GPL-core IgA estimated by enzyme-linked immunosorbent assay (ELISA) have been used for the diagnosis of MAC-PD in clinical practice in Japan since 2011 (Watanabe et al., 2011). Since then, a handful of studies from different countries have been reported on anti-GPL-core IgA-based MAC-PD diagnosis. However, variable sensitivity (48%-85%) and specificity (52% -99%) of anti-core GPL-IgA-based MAC-PD diagnosis were reported in different studies (Kitada et al., 2017; Matsuda et al., 2020; Kawasaki et al., 2022; Choi et al., 2024). It has been noted in a few studies that using a lower diagnostic cut-off value than the manufacturer’s cut-off of anti-core GPL-IgA ELISA kits increases sensitivity and specificity to diagnose MAC-PD (Watanabe et al., 2011; Choi et al., 2024). Overall, varying sensitivities and specificities may be attributable to different diagnostic cut-offs for anti-core GPL-IgA levels, as well as differences in geographic, ethnic, and research population characteristics (Choi et al., 2024). Moreover, factors associated with lower anti-core GPL-IgA levels include immunocompromised states such as HIV/AIDS (patients on prophylactic macrolide monotherapy), cancer, etc. resulting in the possibility of higher false-negative rates. Therefore, different optimal cut-off points of anti-core GPL-IgA levels must be set in these populations (Choi et al., 2024).

**Table 4:** Timeline for biomarkers for NTM disease.

Year	Name of biomarkers	Compartment	Comments	References
1969-2000s (discovery and structural and functional studies) 2011 (used as a biomarker for NTM-PD)	Anti-GPL-core IgA and IgG	Blood plasma/serum	• Rapid test • Both diagnostic and monitoring marker • Minimally invasive • Used for limited NTM species • Variable diagnostic cut-offs • Broad range of sensitivity and specificity	(Schaefer, 1965; Szulga et al., 1966; Birn et al., 1967; Marks et al., 1969; Jenkins et al., 1971; Yoder and Schaefer, 1971; Jenkins and Marks, 1973; Brennan et al., 1978; Meier et al., 1996; Wallace et al., 1996; Sun and Zhang, 1999; Torrelles et al., 2002; Olivier et al., 2017; Griffith et al., 2018; Sharma et al., 2021; Mingora et al., 2023)
2020	Human microRNAs: hsa-miR-484, hsa-miR-584-5p, hsa-miR-625-3p, and hsa-miR-4732-5p	Total RNA (human)	• Serum-specific miRNA signature • Host antibacterial pathway modulators • Costly and requires expertise • Requires to be validated in a large cohort for various NTM species	(Schorey and Sweet, 2008)
2022	2,3,5,8-tetramethyl-decane 2,2,4,6,6-Pentamethyl- heptane 2,3-dimethyl-3-heptene, tridecane and ethanol	Breath	• Study done in CF patients only • Costly and requires expertise • Requires to be validated in a large cohort for various NTM species	(Watanabe et al., 2011)
2023	CRP	Serum	• Prognostic marker • Widely available and cost-effective • Nonspecific	(Matsuda et al., 2020)
2024	HBA1, HBA2, HBD and METTL7B genes	Genomic DNA	• New markers • Requires expertise • Costly • Requires to be validated in a large cohort for various NTM species	(Torrelles et al., 2002)
2024	Methionine, hypoxanthine acetylserotonin adenosine, cystine, and acetylcholine	Urine	• Non-invasive • Experimental stage • Costly and requires expertise • Requires to be validated in a large cohort for various NTM species	(Kitada et al., 2017)

NTM-PD=nontuberculous mycobacterial disease; anti-GPL-core IgA and IgG= anti-glycopeptidolipid-core antibodies [immunoglobulin A (IgA) and immunoglobulin G (IgG)], HBA1 HBA2= hemoglobin-α 1 and 2, HBD=hemoglobin subunit delta, METTL7B=methyltransferase-like 7B, CRP = C-reactive protein, CF= cystic fibrosis, hsa-miR= *Homo sapiens* microRNA.

Some studies also reported that anti-core GPL-IgA levels are useful in detecting *M. abscessus* infection (Manbenmad et al., 2023). It was also observed that the IgA2 subclass of IgA antibodies is predominantly elevated in patients with NTM disease (Steffen et al., 2020). Few studies from Thailand also showed the utility of anti-core GPL-IgG which has a greater specificity than anti-core GPL-IgA in diagnosing NTM-PD. Further, it has been demonstrated that plasma anti-GPL-core IgG levels in patients with SGM were higher than in patients with RGM. Patients with disseminated NTM also exhibit higher elevated anti-core GPL-IgA (Nithichanon et al., 2020).

Recently, high throughput gene sequencing revealed some genetic markers that can differentiate between NTM, TB infection (TBI), and active TB (Schorey and Sweet, 2008). The differential expressions of four hub genes: hemoglobin-α 1 and 2 (HBA1 and HBA2), hemoglobin subunit delta (HBD), and methyltransferase-like 7B (METTL7B) have been shown useful in identifying NTM, LTBI, and active TB patients (Jia et al., 2024). Upregulated HBA1 and 2 in LTBI patients distinguish individuals having NTM disease. Upregulated HBD genes in active TB patients can distinguish them from NTM patients. Moreover, the downregulated METTL7B gene has been associated with LTBI and may distinguish LTBI from active TB (Jia et al., 2024). A South Korean study demonstrated that homo sapiens microRNAs (has-miR) such as hsa-miR-484, hsa-miR-584-5p, hsa-miR-625-3p, and hsa-miR-4732-5p are differentially expressed in individuals with NTM-PD and may serve as possible biomarkers for the disease (Han et al., 2020). A Canadian study on cystic fibrosis discovered that breath samples from patients with NTM-PD had differential levels of volatile molecules. When compared to healthy controls, NTM-PD patients had significantly higher levels of these compounds, including 2,3,5,8-tetramethyl-decane, 2,2,4,6,6-pentamethyl-heptane, 2,3-dimethyl-3-heptene, and tridecane, but ethanol levels were significantly lower (Mani-Varnosfaderani et al., 2022). A South Korean study revealed urine biomarkers that can differentiate between NTM and TB disease (Anh et al., 2024). These biomarkers included methionine, hypoxanthine, and acetylserotonin, which were all 1.8-fold elevated in NTM patients (Anh et al., 2024). In contrast, the level of 4-guanidinobutanoate decreased nearly threefold, while adenosine, cystine, and acetylcholine decreased approximately 1.7fold in NTM patients (Anh et al., 2024).

There are very few studies on prognostic markers for NTM disease. A South Korean study (Park et al., 2023) showed that elevated serum C-reactive protein (CRP) levels at diagnosis predicted a worse prognosis in individuals with non-tuberculous mycobacterial pulmonary disease (NTM-PD). Approximately one-fourth of NTM-PD patients had elevated CRP levels, which increased their risk of death (Park et al., 2023). Data from Korea (Koh, et al., 2012) suggest that *M. intracellulare* are more virulent and are associated with a worse prognosis. NTM disease with *M. avium* and *M. intracellulare* behave similarly clinically and therapeutic response wise. *M. avium* often presents with disseminated disease (Koh, et al., 2012).

## 7 Conclusion

NTM has undertaken a rather very long journey to reach its current status, from anonymity to intense research efforts are on to unravel several areas, such as rapid diagnosis, independent of culture, development of new biomarkers for early diagnosis, and monitoring treatment response. Equal importance is being given to drug discovery for the development of new drugs for an effective NTM treatment. In spite of a heightened degree of awareness about NTM diseases among microbiologists and clinicians in India more efforts are required to determine the exact species/subspecies of isolated NTM and other high TB burden countries disease prevalence including geographical variations and treatment outcomes. The measures should be taken by various stakeholders to formulate India-centric guidelines for diagnosis and treatment of NTM disease and eventually inclusion in the national healthcare programmes of India. Efforts should be made to establish a National Registry of NTM in India.

## References

[B1] Alvarez G. Tavel R. (1885). Recherches sur le bacille de Lustgarten. Arch. Physiol. Norm Pathol. 3, 303–306.

[B2] Anh N. K. Phat N. K. Thu N. Q. Tien N. T.N. Eunsu C. Kim H. S. . (2024). Discovery of urinary biosignatures for tuberculosis and nontuberculous mycobacteria classification using metabolomics and machine learning. Sci. Rep. 14, 15312. doi: 10.1038/s41598-024-66113-x, PMID: 38961191 PMC11222504

[B3] Anonymous (1990). Diagnosis and treatment of disease caused by nontuberculous mycobacteria. Am. Rev. Respir. Dis. 142, 940–953. doi: 10.1164/ajrccm/142.4.940, PMID: 2282111

[B4] Anonymous (1997). Diagnosis and treatment of disease caused by nontuberculous mycobacteria. This official statement of the American Thoracic Society was approved by the Board of Directors, March 1997. Medical Section of the American Lung Association. Am. J. Respir. Crit. Care Med. 156, S1–25. doi: 10.1164/ajrccm.156.2.atsstatement, PMID: 9279284

[B5] Aronson J. D. (1926). Spontaneous tuberculosis in salt water fish. J. Infect. Dis. 39, 315–320. doi: 10.1093/infdis/39.4.315

[B6] Arvind B. Medigeshi G. R. Kapil A. Xess I. Singh U. Lodha R. . (2020). Aetiological agents for pulmonary exacerbations in children with cystic fibrosis: An observational study from a tertiary care centre in northern India. Indian J. Med. Res. 151, 65–70. doi: 10.4103/ijmr.IJMR_1275_18, PMID: 32134016 PMC7055172

[B7] Becker G. J. Walker R. G. Dziukas L. J. Harvey K. J. Valentine R. Kincaid-Smith P . (1980). Renal infection with *Mycobacterium chelonei*. Aust. N Z J. Med. 10, 44–47. doi: 10.1111/j.1445-5994.1980.tb03417.x, PMID: 6929675

[B8] Bessesen M. T. Berry C. D. Iohnson M. A. Klaus B. Blaser M. J. Ellison R. T . (1990). “ Site of origin of disseminated MAC infectionin AIDS [abstract 1268],” in Program and abstracts of the 30th interscience conference on antimicrobial agents and chemotherapy (Atlanta) ( American Society for Microbiology, Washington, DC).

[B9] Bhattacharya S. Banik A. Majumdar T. Das B. (2021). Cutaneous microabscesses by *mycobacterium chelonae*: case reports from a tertiary care centre, Tripura. Int. J. Adv. Res. 9, 713–716. doi: 10.21474/IJAR01/13600

[B10] Birn K. J. Schaefer W. B. Jenkins P. A. Szulga T. Marks J. (1967). Classification of *Mycobacterium avium* and related opportunist mycobacteria met in England and Wales. J. Hyg (Lond) 65, 575–589. doi: 10.1017/S0022172400046106, PMID: 4864326 PMC2130390

[B11] Bojalil L. F. Cerbon J. Trujillo A. (1962). Adansonian classification of mycobacteria. J. Gen. Microbiol. 28, 333–346. doi: 10.1099/00221287-28-2-333, PMID: 13870716

[B12] Branch A. (1933). A study of acid-fast organisms other than mammalian tubercle bacilli isolated from disease in man: the occurrence of avian tubercle bacillus infection of men and the diagnosis of avirulent avian tubercle bacilli. Tubercle 14, 337–353. doi: 10.1016/S0041-3879(33)80110-3

[B13] Brennan P. J. Souhrada M. Ullom B. McClatchy J. K. Goren M. B . (1978). Identification of atypical mycobacteria by thin-layer chromatography of their surface antigens. J. Clin. Microbiol. 8, 374–379. doi: 10.1128/jcm.8.4.374-379.1978, PMID: 721943 PMC275256

[B14] Brown-Elliott B. A. Philley J. V. (2017). Rapidly growing mycobacteria. Microbiol. Spectr. 5. doi: 10.1128/microbiolspec.TNMI7-0027-2016, PMID: 28084211 PMC11687460

[B15] Brown-Elliott B. A. Wallace R. J. Jr Blinkhorn R. . (2001). Successful treatment of disseminated *Mycobacterium chelonae* infection with linezolid. Clin. Infect. Dis. 33, 1433–1434. doi: 10.1086/322523, PMID: 11550122

[B16] Buhler V. B. Pollak A. (1953). Human infection with atypical acid-fast organisms; report of two cases with pathologic findings. Am. J. Clin. Pathol. 23, 363–374. doi: 10.1093/ajcp/23.4.363, PMID: 13040295

[B17] Carruthers K. J. Edwards F. G. (1965). Atypical mycobacteria in western Australia. Am. Rev. Respir. Dis. 91, 887–895. doi: 10.1164/arrd.1965.91.6.887, PMID: 14294712

[B18] Chancharoenthana W. Kamolratanakul S. Rotcheewaphan S. Leelahavanichkul A. Schultz M. J. (2025). Recent advances in immunopathogenesis and clinical practice: Managing non-tuberculous mycobacteria. Front. Immunol. 16, 1554544. doi: 10.3389/fimmu.2025.1554544, PMID: 40176807 PMC11961655

[B19] Choi H. Hughes C. Eke Z. Shuttleworth M. Shteinberg M. Polverino E. . (2024). Clinical efficacy of serum antiglycopeptidolipid core IgA antibody test for screening nontuberculous mycobacterial pulmonary disease in bronchiectasis: a european multicenter cohort study. Chest S0012-3692, 05418–05417. doi: 10.1016/j.chest.2024.10.029, PMID: 39490969 PMC12106963

[B20] Choudhri D. S. Dube M. K. Purohit S. D. Dube S. (1979). The prevalence of anonymous mycobaeteria in both resistant as well as fresh cases of pulmonary tuberculosis in the local population of south east Rajasthan. Indian J. Pathol. Microbiol. 22, 162–175., PMID: 114478

[B21] Clinical and Laboratory Standards Institute (2018). Susceptibility testing of mycobacteria, Nocardiae, and other aerobic actinomycetes. 3rd edition (Wayne, PA: Clinical and Laboratory Standards Institute). 31339680

[B22] Cobbett L. (1918). An acid-fast bacillus obtained from a pustular eruption. Br. Med. J. 2, 158–159. doi: 10.1136/bmj.2.3007.158-a, PMID: 20769139 PMC2341667

[B23] Crow H. E. King C. T. Smith C. E. Corpe R. F. Stergus I . (1957). A limited clinical, pathologic, and epidemiologic study of patients with pulmonary lesions associated with atypical acid-fast bacilli in the sputum. Am. Rev. Tuberc 75, 199–222. doi: 10.1164/artpd.1957.75.2.199, PMID: 13403152

[B24] CruzAguilar M. CastilloRodal A. I. ArredondoHernández R. LópezVidal Y. (2021). Non-tuberculous mycobacteria immunopathogenesis: Closer than they appear—A prime of innate immunity trade-off and NTM ways into virulence. Scand. J. Immunol. 94, e13035. doi: 10.1111/sji.13035, PMID: 33655533 PMC9285547

[B25] Cummins S. L. Williams E. M. (1933). An “acid-fast” other than Koch’s bacillus cultivated from sputum. Tubercle 15, 49–53. doi: 10.1016/S0041-3879(33)80019

[B26] Cuttino J. T. McCabe A. M. (1949). Pure granulomatous nocardiosis: A new fungus disease distinguished by intracellular parasitism. A description of a new disease in man due to a hitherto undescribed organism, Nocardia intracellularis, n. sp., including a study of the biologic and pathogenic properties of this species. Am. J. Pathol. 25, 1–47., PMID: 18106962 PMC1942779

[B27] da Costa Cruz J. C. (1938). *Mycobacterium fortuitum*: um novo bacilo acido-resistente patogenico para o homen (new acid fast bacillus pathogenic for man). Acta Med. Rio Janeiro 1, 298–301.

[B28] Dadheech M. Malhotra A. G. Patel S. Singh J. Khadanga S. Khurana A. . (2023). Molecular identification of non-tuberculous mycobacteria in suspected tuberculosis cases in central India. Cureus 15, e39992. doi: 10.7759/cureus.39992, PMID: 37416024 PMC10321564

[B29] Daley C. L. Iaccarino J. M. Lange C. Cambau E. Wallace R. J. Jr Andrejak C. . (2020). Treatment of nontuberculous mycobacterial pulmonary disease: an official ATS/ERS/ESCMID/IDSA clinical practice guideline. Eur. Respir. J. 56, 2000535. doi: 10.1183/13993003.00535-2020, PMID: 32636299 PMC8375621

[B30] Dalovisio J. R. Pankey G. A. Wallace R. J. Jones D. B. (1981). Clinical usefulness of amikacin and doxycycline in the treatment of infection due to *Mycobacterium fortuitum* and *Mycobacterium chelonei*. Rev. Infect. Dis. 3, 1068–1074. doi: 10.1093/clinids/3.5.1068, PMID: 7339806

[B31] Das S. Mishra B. Mohapatra P. R. Preetam C. Rath S. (2022). Clinical presentations of nontuberculous mycobacteria as suspected and drug-resistant tuberculosis: Experience from a tertiary care center in Eastern India. Int. J. Mycobacteriol 11, 167–174. doi: 10.4103/ijmy.ijmy_68_22, PMID: 35775549

[B32] Dedrick R. M. Guerrero Bustamante C. A. Garlena R. A. Pinches R. S. Cornely K. Hatfull G. F . (2019). Mycobacteriophage ZoeJ: a broad host-range close relative of mycobacteriophage TM4. Tuberculosis (Edinb) 115, 14–23. doi: 10.1016/j.tube.2019.01.002, PMID: 30948168 PMC6452893

[B33] De Mandal S. Panda A. K. Lalnunmawii E. Bisht S. S. Kumar N. S . (2015). Illumina-based analysis of bacterial community in Khuangcherapuk cave of Mizoram, Northeast India. Genom Data 5, 13–14. doi: 10.1016/j.gdata.2015.04.023, PMID: 26484212 PMC4583610

[B34] Department of AIDS Control Ministry of Health and Family Welfare Government of India National framework for joint HIV/TB collaborative activities, November 2013. Available online at: https://naco.gov.in/sites/default/files/National%20Framework%20for%20Joint%20HIV%20TB%20Collaborative%20Activities%20November%20%202%20%281%29.pdf (Accessed July 1, 2025).

[B35] Floto R. A. Olivier K. N. Saiman L. Daley C. L. Herrmann J. L. Nick J. A. . (2016). US Cystic Fibrosis Foundation and European Cystic Fibrosis Society. US Cystic Fibrosis Foundation and European Cystic Fibrosis Society consensus recommendations for the management of non-tuberculous mycobacteria in individuals with cystic fibrosis. Thorax 71 Suppl 1(Suppl 1), i1–22. 26666259 10.1136/thoraxjnl-2015-207360 PMC4717371

[B36] Garima K. Varma-Basil M. Pathak R. Kumar S. Narang A. Rawat K. S. . (2012). Are we overlooking infections owing to non-tuberculous mycobacteria during routine conventional laboratory investigations? Int. J. Mycobacteriol 1, 207–211. doi: 10.1016/j.ijmyco.2012.10.005, PMID: 26785625

[B37] Gibson J. B. (1953). Infection of the lungs by “saprophytic” mycobacteria in achalasia of the cardia, with report of a fatal case showing lipoid pneumonia due to milk. J. Pathol. Bacteriol. 65, 239–251. doi: 10.1002/path.1700650125, PMID: 13035617

[B38] Griffith D. E. Aksamit T. Brown-Elliott B. A. Catanzaro A. Daley C. Gordin F. . (2007). ATS Mycobacterial Diseases Subcommittee; American Thoracic Society; Infectious Disease Society of America. An official ATS/IDSA statement: Diagnosis, treatment, and prevention of nontuberculous mycobacterial diseases. Am. J. Respir. Crit. Care Med. 175, 367–416. doi: 10.1164/rccm.200604-571ST, PMID: 17277290

[B39] Griffith D. E. Brown-Elliott B. A. Wallace R. J. Jr. (2003). Thrice-weekly clarithromycin-containing regimen for treatment of *Mycobacterium kansasii* lung disease: results of a preliminary study. Clin. Infect. Dis. 37, 1178–1182. doi: 10.1086/378742, PMID: 14557961

[B40] Griffith D. E. Eagle G. Thomson R. Aksamit T. R. Hasegawa N. Morimoto K. . (2018). Amikacin liposome inhalation suspension for treatment-refractory lung disease caused by *Mycobacterium avium* complex (CONVERT). A prospective, open-label, randomized study. Am. J. Respir. Crit. Care Med. 198, 1559–1569. doi: 10.1164/rccm.201807-1318OC, PMID: 30216086

[B41] Gupta P. Katoch V. M. Gupta U. D. Chauhan D. S. Das R. Singh D. . (2002). A preliminary report on characterization and identification of non tuberculous mycobacteria (NTM) on the basis of biochemical tests and protein/isoenzyme electrophoretic patterns. Indian J. Med. Microbiol. 20, 137–140. doi: 10.1016/S0255-0857(21)03245-X, PMID: 17657052

[B42] Gupta N. Mittal A. Niyas V. K. M. Banerjee S. Ray Y. Kodan P. . (2020). Nontuberculous mycobacteria: A report of eighteen cases from a tertiary care center in India. Lung India 37, 495–500. doi: 10.4103/lungindia.lungindia_365_19, PMID: 33154211 PMC7879861

[B43] Han S. A. Jhun B. W. Kim S. Y. Moon S. M. Yang B. Kwon O. J. . (2020). miRNA Expression Profiles and potential as biomarkers in nontuberculous mycobacterial pulmonary disease. Sci. Rep. 10, 3178. doi: 10.1038/s41598-020-60132-0, PMID: 32081976 PMC7035291

[B44] Haworth C. S. Banks J. Capstick T. F. Fisher A. J. Gorsuch T. Laurenson I. F. . (2017). British Thoracic Society guidelines for the management of non-tuberculous mycobacterial pulmonary disease (NTM-PD). Thorax 72, ii1–i64. doi: 10.1136/thoraxjnl-2017-210929, PMID: 29054853

[B45] Hegde N. R. Talari M. Majumdar S. S. (2024). One Health initiative in India: Genesis and hurdles in establishing the first consortium. Vet. World. 17, 2925–2931. doi: 10.14202/vetworld.2024.2925-2931, PMID: 39897352 PMC11784042

[B46] Jain N. K. Kachroo B. B. Prasad G. Hanifa M. F . (1991). Identification and characterisation of mycobacteria other than TB by protein finger printing. Indian J. Tuberc 38, 91–94.

[B47] Jain S. Sankar M. M. Sharma N. Singh S. Chugh T. D . (2014). High prevalence of non-tuberculous mycobacterial disease among non-HIV infected individuals in a TB endemic country–experience from a tertiary center in Delhi, India. Pathog. Glob Health 108, 118–122. doi: 10.1179/2047773214Y.0000000133, PMID: 24649868 PMC4005591

[B48] Jani M. N. Rodrigues C. S. Mehta A. P. (2011). The neglected and often ignored: nontuberculous mycobacteria. J. Glob Infect. Dis. 3, 94. doi: 10.4103/0974-777X.77305, PMID: 21572618 PMC3068589

[B49] Jenkins P. A. Marks J. (1973). Thin-layer chromatography of mycobacterial lipids as an aid to classification. Ann. Soc. Belg Med. Trop. 53, 331–337. doi: 10.1164/arrd.1971.103.2.179, PMID: 4198567

[B50] Jenkins P. A. Marks J. Schaefer W. B. (1971). Lipid chromatography and seroagglutination in the classification of rapidly growing mycobacteria. Am. Rev. Respir. Dis. 103, 179–187., PMID: 5541466 10.1164/arrd.1971.103.2.179

[B51] Jesudason M. V. Gladstone P. (2005). Non tuberculous mycobacteria isolated from clinical specimens at a tertiary care hospital in south India. Indian J. Med. Microbiol. 23, 172–175. doi: 10.1016/S0255-0857(21)02588-3, PMID: 16100423

[B52] Jia Q. Wu Y. Huang Y. Bai X . (2024). New genetic biomarkers from transcriptome RNA-sequencing for Mycobacterium tuberculosis complex and *Mycobacterium avium* complex infections by bioinformatics analysis. Sci. Rep. 14, 17385. doi: 10.1038/s41598-024-68242-9, PMID: 39075154 PMC11286745

[B53] Jones J. S. (1969). *Mycobacterium kansasii* in East Kent: A report of seven pulmonary infections with an environmental study. Br. J. Dis. Chest 63, 83–95. doi: 10.1016/s0007-0971(69)80033-1, PMID: 5771897

[B54] Kalita J. B. Rahman H. Baruah K. C. (2005). Delayed post-operative wound infections due to non-tuberculous Mycobacterium. Indian J. Med. Res. 122, 535–539., PMID: 16518006

[B55] Karak K. Bhattacharyya S. Majumdar S. De P. K . (1996). Pulmonary infection caused by mycobacteria other than M. tuberculosis in and around Calcutta. Indian J. Pathol. Microbiol. 39, 131–134., PMID: 9401242

[B56] Karlson A. G. Feldman W. H. (1953). Mycobacteria of human origin resembling *Mycobacterium avium*. Proc. 15th Int. Veterinary Congress Stockholm 1, 159.

[B57] Kawasaki T. Kitada S. Fukushima K. Akiba E. Haduki K. Saito H. . (2022). The diagnosis of nontuberculous mycobacterial pulmonary disease by single bacterial isolation plus anti-GPL-core IgA antibody. Microbiol. Spectr. 10, e0140621. doi: 10.1128/spectrum.01406-21, PMID: 34985326 PMC8729764

[B58] Khatter S. Singh U. B. Arora J. Rana T. Seth P . (2008). Mycobacterial infections in human immuno-deficiency virus seropositive patients: role of non-tuberculous mycobacteria. Indian J. Tuberc 55, 28–33., PMID: 18361308

[B59] Kim B. G. Kim H. Kwon O. J. Huh H. J. Lee N. Y. Baek S-Y. . (2020). Outcomes of inhaled amikacin and clofazimine-containing regimens for treatment of refractory *Mycobacterium avium* complex pulmonary disease. J. Clin. Med. 9, 2968. doi: 10.3390/jcm9092968, PMID: 32937940 PMC7565500

[B60] Kim Y. J. Sapkota A. Lee B. Park E. J. Jo E. K. (2025). The role of autophagy during nontuberculous mycobacterial infection. J. Bacteriol Virol. 55, 205–221. doi: 10.4167/jbv.2025.55.3.205

[B61] Koh W. J. Jeong B. H. Jeon K. Lee N. Y. Lee K. S. Woo S. Y. . (2012). Clinical significance of the differentiation between *Mycobacterium avium* and *Mycobacterium intracellulare* in *M. avium* complex lung disease. Chest 142, 1482–1488. doi: 10.1378/chest.12-0494, PMID: 22628488

[B62] Kitada S. Maekura R. Yoshimura K. Miki K. Miki M. Oshitani Y. . (2017). Levels of antibody against glycopeptidolipid core as a marker for monitoring treatment response in *Mycobacterium avium* complex pulmonary disease: a prospective cohort study. J. Clin. Microbiol. 55, 884–892. doi: 10.1128/JCM.02010-16, PMID: 28031437 PMC5328456

[B63] Kotian M. Ganesan V. Sarvamangala J. N. Shivananda P. G. Achyutha K. N . (1981). Pulmonary infections by atypical mycobacteria in a rural coastal region of Karnataka, India. Trop. Geogr. Med. 33, 117–121., PMID: 7281210

[B64] Kusunoki S. Ezaki T. (1992). Proposal of Mycobacterium peregrinum sp. nov., nom. rev., and elevation of *mycobacterium chelonae* subsp. abscessus (Kubica et al.) to species status: *Mycobacterium abscessus* comb. nov. Int. J. Syst. Bacteriol 42, 240–245. doi: 10.1099/00207713-42-2-240, PMID: 1581184

[B65] Lange C. Böttger E. C. Cambau E. Griffith D. E. Guglielmetti L. van Ingen J. . (2022). Expert panel group for management recommendations in nontuberculous mycobacterial pulmonary diseases. Lancet Infect. Dis. 22, e178–e190. doi: 10.1016/S1473-3099(21)00586-7, PMID: 35090639

[B66] Lominski I. Harper E. M. (1953). An unidentified acid-fast bacillus (Mycobacterium sp. strain Glasgow) found in human sputum and lung lesions. J. Pathol. Bacteriol 65, 253–254. doi: 10.1002/path.1700650126, PMID: 13035618

[B67] Lunn H. F. Rees R. J. (1964). Treatment of mycobacterial skin ulcers in Uganda with a riminophenazine derivative (B. 663). Lancet 283, 247–249. doi: 10.1016/S0140-6736(64)92351-7, PMID: 14086214

[B68] Lyu J. (2024). Immunopathogenesis of non-tuberculous mycobacteria lung disease. Korean J. Med. 99, 169–176. doi: 10.3904/kjm.2024.99.4.169

[B69] MacCallum P. Tolhurst J. C. Buckle G. Sissons H. A. (1948). A new mycobacterial infection in man. I. Clinical aspects. II. Experimental investigations in laboratory animals. III. Pathology of the experimental lesions in the rat. IV. Cultivation of the new mycobacterium. J. Pathol. Bacteriol 60, 93–122. doi: 10.1002/path.1700600111

[B70] Maclean J. T. Smith F. (1947). Use of streptomycin in non-tuberculous urinary tract infections: (Preliminary Report). Can. Med. Assoc. J. 57, 131–136. PMC1590607 20253846

[B71] Manbenmad V. So-Ngern A. Chetchotisakd P. Faksri K. Ato M. Nithichanon A. . (2023). Evaluating anti-GPL-core IgA as a diagnostic tool for non-tuberculous mycobacterial infections in Thai patients with high antibody background. Sci. Rep. 13, 18883. doi: 10.1038/s41598-023-45893-8, PMID: 37919326 PMC10622420

[B72] Mani-Varnosfaderani A. Gao A. Poch K. R. Caceres S. M. Nick J. A. Hill J. E . (2022). Breath biomarkers associated with nontuberculous mycobacterial disease status in persons with cystic fibrosis: a pilot study. J. Breath Res. 16. doi: 10.1088/1752-7163/ac6bb6, PMID: 35487186

[B73] Marks J. Jenkins P. A. Schaefer W. B. (1969). Identification and incidence of a third type of *Mycobacterium avium*. Tubercle 50, 394–395. doi: 10.1016/0041-3879(69)90040-3, PMID: 5392110

[B74] Marzinowski E. J. (1900). Uber einige in den Krypt en der gaumenmandeln gefundene Bacillenarten. Zentralbl Mikrobiol 28, 39–45.

[B75] Matsuda S. Asakura T. Morimoto K. Suzuki S. Fujiwara K. Furuuchi K. . (2020). Clinical significance of anti-glycopeptidolipid-core IgA antibodies in patients newly diagnosed with *Mycobacterium avium* complex lung disease. Respir. Med. 171, 106086. doi: 10.1016/j.rmed.2020.106086, PMID: 32917357

[B76] Maurya A. K. Nag V. L. Kant S. Kushwaha R. A. Kumar M. Singh A. K. . (2015). Prevalence of nontuberculous mycobacteria among extrapulmonary tuberculosis cases in tertiary care centers in Northern India. BioMed. Res. Int. 2015, 465403. doi: 10.1155/2015/465403, PMID: 25883962 PMC4391508

[B77] Meier A. Heifets L. Wallace R. J. Jr Zhang Y. Brown B. A. Sander P. . (1996). Molecular mechanisms of clarithromycin resistance in *Mycobacterium avium*: observation of multiple 23S rDNA mutations in a clonal population. J. Infect. Dis. 174, 354–360. doi: 10.1093/infdis/174.2.354, PMID: 8699066

[B78] Mingora C. M. Bullington W. Faasuamalie P. E. Levin A. Porter G. Stadnick R. . (2023). Long-term safety and tolerability of omadacycline for the treatment of *Mycobacterium abscessus* infections. Open Forum Infect. Dis. 10, ofad335. doi: 10.1093/ofid/ofad335, PMID: 37476076 PMC10354853

[B79] Mishra P. S. Narang P. Narang R. Goswami B. Mendiratta D. K. (2018). Spatio-temporal study of environmental nontuberculous mycobacteria isolated from Wardha district in Central India. Antonie Van Leeuwenhoek 111, 73–87. doi: 10.1007/s10482-017-0927-2, PMID: 28836034

[B80] Moore M. Frerichs J. B. (1953). An unusual acid-fast infection of the knee with subcutaneous, abscess-like lesions of the gluteal region: Report of a case with a study of the organism, *Mycobacterium abscessus*, n. sp. J. Invest. Dermatol. 20, 133–169. doi: 10.1038/jid.1953.18, PMID: 13035193

[B81] Mudliar S. K. R. Kulsum U. Rufai S. B. Umpo M. Nyori M. Singh S . (2022). Snapshot of mycobacterium tuberculosis phylogenetics from an Indian state of Arunachal pradesh bordering China. Genes (Basel) 13, 263. doi: 10.3390/genes13020263, PMID: 35205308 PMC8872330

[B82] Mustafa A. S. Talwar G. P. (1978). Early and late reactions in tuberculoid and lepromatous leprosy patients with lepromins from *Mycobacterium leprae* and five selected cultivable mycobacteria. Lepr India 50, 566–571., PMID: 374875

[B83] Narain R. Chandrasekhar P. Satyanarayanachar R. A. Lal P . (1968). Resistant and sensitive strains of Mycobacterium tuberculosis found in repeated surveys among a South Indian rural population. Bull. World Health Organ 39, 681–699., PMID: 4978410 PMC2554435

[B84] Narang R. Narang P. Mendiratta D. K. (2009). Isolation and identification of nontuberculous mycobacteria from water and soil in central India. Indian J. Med. Microbiol. 27, 247–250. doi: 10.4103/0255-0857.53208, PMID: 19584507

[B85] Nithichanon A. Samer W. Chetchotisakd P. Kewcharoenwong C. Ato M. Lertmemongkolchai G . (2020). Evaluation of plasma anti-GPL-core IgA and IgG for diagnosis of disseminated non-tuberculous mycobacteria infection. PloS One 15, e0242598. doi: 10.1371/journal.pone.0242598, PMID: 33253290 PMC7703992

[B86] Norden A. Linell F. (1951). A new type of pathogenic mycobacterium. Nature 168, 826. doi: 10.1038/168826a0, PMID: 14890762

[B87] Nontuberculous mycobacteria (overview). Available online at: https://www.nationaljewish.org/conditions/ntm-nontuberculous-mycobacteria/ntm-nontuberculous-mycobacteria-overview/history (Accessed July 19, 2024).

[B88] Olivier K. N. Griffith D. E. Eagle G. McGinnis J. P. Micioni L. 2nd Liu K. . (2017). Randomized trial of liposomal amikacin for inhalation in nontuberculous mycobacterial lung disease. Am. J. Respir. Crit. Care Med. 195, 814–823. doi: 10.1164/rccm.201604-0700OC, PMID: 27748623 PMC5363966

[B89] Ophüls W. (1904). Chronic subcutaneous abscess in man containing acid-proof bacilli in pure culture. J. Med. Res. 11, 439–443., PMID: 19971611 PMC2101837

[B90] Palmer C. E. (1953). Tuberculin sensitivity and contact with tuberculosis: Further evidence of nonspecific sensitivity. Am. Rev. Tuberc 68, 678–694. doi: 10.1164/art.1953.68.5.678, PMID: 13104882

[B91] Panel on opportunistic infections in adults and adolescents with HIV. Guidelines for the prevention and treatment of opportunistic infections in adults and adolescents with HIV: recommendations from the centers for disease control and prevention, the national institutes of health, and the HIV medicine association of the infectious diseases society of America. Available online at: http://aidsinfo.nih.gov/contentfiles/lvguidelines/adult_oi.pdf (Accessed July 19, 2024).

[B92] Paramasivan C. N. Govindan D. Prabhakar R. Somasundaram P. R. Subbammal S. Tripathy S. P . (1985). Species level identification of non-tuberculous mycobacteria from South Indian BCG trial area during 1981. Tubercle 66, 9–15. doi: 10.1016/0041-3879(85)90048-0, PMID: 3984041

[B93] Parashar D. Das R. Chauhan D. S. Sharma V. D. Lavania M. Yadav V. S. . (2009). Identification of environmental mycobacteria isolated from Agra, north India by conventional & molecular approaches. Indian J. Med. Res. 129, 424–431., PMID: 19535838

[B94] Park H. J. Kim J. Y. Yim J. J. Kwak N . (2023). Prognostic serum biomarkers in non-tuberculous mycobacterial pulmonary disease. J. Infect. Chemother. 29, 1005–1007. doi: 10.1016/j.jiac.2023.06.017, PMID: 37385407

[B95] Pereira A. C. Ramos B. Reis A. C. Cunha M. V . (2020). Non-tuberculous mycobacteria: molecular and physiological bases of virulence and adaptation to ecological niches. Microorganisms 9, 8:1380. doi: 10.3390/microorganisms8091380, PMID: 32916931 PMC7563442

[B96] Philley J. V. Wallace R. J. Jr. Benwill J. L. Taskar V. Brown-Elliott B. A. Thakkar F. . (2015). Preliminary results of bedaquiline as salvage therapy for patients with nontuberculous mycobacterial lung disease. Chest 148, 499–506. doi: 10.1378/chest.14-2764, PMID: 25675393 PMC4694173

[B97] Radha Bai Prabhu T. Pandiyan N. Sujatha N. Jawahar M. S . (2019). Significance of isolating non-tuberculous mycobacterial organisms in infertile women with tubal disease: an observational study. BJOG 126 Suppl 4, 66–71. doi: 10.1111/1471-0528.15814, PMID: 31074566

[B98] Ramakrishnan C. V. (1981). Pulmonary disease due to atypical mycobacteria: a retrospective study from South India. Rev. Infect. Dis. 3, 1090–1092. doi: 10.1093/clinids/3.5.1090, PMID: 7339810

[B99] Rao K. N. Kotian M. (1976). Atypical mycobacteria and sterility. Lancet 1, 1301–1302. doi: 10.1016/S0140-6736(76)91778-5, PMID: 73732

[B100] Rastogi N. Goh K. S. Bryskier A. Devallois A . (1996). Spectrum of activity of levofloxacin against nontuberculous mycobacteria and its activity against the *Mycobacterium avium* complex in combination with ethambutol, rifampin, roxithromycin, amikacin, and clofazimine. Antimicrob. Agents Chemother. 40, 2483–2487. doi: 10.1128/AAC.40.11.2483, PMID: 8913450 PMC163561

[B101] Ravindran B. Hennessy D. O’Hara M. Tay E. L. Banuve R. S. McVernon J. . (2025). Epidemiology of buruli ulcer in victoria, Australia, 2017-2022. Emerg. Infect. Dis. 31, 448–457. doi: 10.3201/eid3103.240938, PMID: 40023793 PMC11878321

[B102] Rodrigues C. (2015). The expanding repertoire of non tuberculous mycobacterial infections: focus on rapidly growing mycobacteria bloodstream infections. J. Assoc. Physicians India 63, 9–10., PMID: 26591120

[B103] Rolfe N. E. Toney J. F. Green M. R. Sandin R. L. Greene J. N . (2007). Successful treatment of *Mycobacterium fortuitum* osteomyelitis after allogeneic bone marrow transplantation. Infect. Dis. Clin. Pract. 15, 1339–1340. doi: 10.1097/IPC.0b013e31802df51b

[B104] Rothstein E. (1969). The twenty-eighth veterans administration—Armed forces pulmonary disease research conference. Am. Rev. Respir. Dis. 99, 804–819. 10.1164/arrd.1964.90.1.129 14178620

[B105] Runyon E. H. (1955). Veterans Administration-National Tuberculosis Association cooperative study of mycobacteria. Am. Rev. Tuberc Pulm Dis. 72, 866–870.

[B106] Runyon E. H. (1959). Anonymous mycobacteria in pulmonary disease. Med. Clin. North Am. 43, 273–290. doi: 10.1016/S0025-7125(16)34193-1, PMID: 13612432

[B107] Sali G. Kalavampara S. V. Kandasamy S. G. Kumar A. Mathew G. Thomas A . (2021). Genitourinary non-tuberculous mycobacterial (GU-NTM) infections: A single institution experience in South India. Indian J. Tuberc 68, 65–72. doi: 10.1016/j.ijtb.2020.08.018, PMID: 33641853

[B108] Satta Y. Yamashita M. Matsuo Y. Kiyokawa H. Sato Y. Takemura H. . (2020). Non-tuberculous mycobacterial pseudo-outbreak of an intestinal culture specimen caused by a water tap in an endoscopy unit. Intern Med 59, 2811–2815. doi: 10.2169/internalmedicine.5188-20, PMID: 32641662 PMC7725637

[B109] Schaefer W. B. (1965). Serologic identification and classification of the atypical mycobacteria by their agglutination. Am. Rev. Respir. Dis. 92, 85–93. doi: 10.1164/arrd.1965.92.6P2.85, PMID: 5843844

[B110] Schorey J. S. Sweet L. (2008). The mycobacterial glycopeptidolipids: structure, function, and their role in pathogenesis. Glycobiology 18, 832–841. doi: 10.1093/glycob/cwn076, PMID: 18723691 PMC2733780

[B111] Scott J. P. Ji Y. Kannan M. Wylam M. E . (2018). Inhaled granulocyte-macrophage colony-stimulating factor for *Mycobacterium abscessus* in cystic fibrosis. Eur. Respir. J. 51, 1702127. doi: 10.1183/13993003.02127-2017, PMID: 29419443

[B112] Sebastian G. Nagaraja S. B. Vishwanatha T. Voderhobli M. Vijayalakshmi N. Kumar P . (2018a). Non-Tuberculosis mycobacterium speciation using HPLC under Revised National TB Control Programme (RNTCP) in India. J. Appl. Microbiol. 124, 267–273. doi: 10.1111/jam.13604, PMID: 28990320

[B113] Sebastian G. Nagaraja S. B. Vishwanatha T. Hemalatha K. Vijayalakshmi N. Kumar P . (2018b). Identification of Non-Tuberculous Mycobacterium by LPA (CM/AS) assay, HPLC and biochemical test: which is feasible for RNTCP? Indian J. Tuberc 65, 329–334. doi: 10.1016/j.ijtb.2018.08.003, PMID: 30522621

[B114] Shanker S. V. Jain N. K. Chandrasekhar S. Singh M. M . (1989). Prevalence of atypical mycobacteria in sputum of patients undergoing treatment at a tuberculosis clinic. Indian J. Chest Dis. Allied Sci. 31, 9–13., PMID: 2807421

[B115] Sharma S. K. Sharma R. Singh B. K. Upadhyay V. Mani I. Tripathi M. . (2019). A prospective study of non-tuberculous mycobacterial disease among tuberculosis suspects at a tertiary care centre in north India. Indian J. Med. Res. 150, 458–467. doi: 10.4103/ijmr.IJMR_194_19, PMID: 31939389 PMC6977370

[B116] Sharma S. K. Upadhyay V. (2020). Epidemiology, diagnosis & treatment of non-tuberculous mycobacterial diseases. Indian J. Med. Res. 152, 185–226. doi: 10.4103/ijmr.IJMR_902_20, PMID: 33107481 PMC7881820

[B117] Sharma S. K. Upadhyay V. (2021). Non-tuberculous mycobacteria: a disease beyond TB and preparedness in India. Expert Rev. Respir. Med. 15, 949–958. doi: 10.1080/17476348.2021.1925545, PMID: 33938343

[B118] Sharma S. K. Upadhyay V. Dewan R. Verma A. Ranjan A. Yadav R. . (2025). Prevalence and geographical distribution of various nontuberculous mycobacterial (NTM) species/subspecies in India: Early findings of Indian Council of Medical Research (ICMR), New Delhi Sponsored Multicentre Research Project Between 2021-2024. Am. J. Respir. Crit. Care Med. 211, A7265. doi: 10.1164/ajrccm.2025.211.Abstracts.A7265

[B119] Sharma S. K. Upadhyay V. Mohan A. (2021). What is new in BTS 2017 & ATS/ERS/ESCMID/IDSA 2020 guidelines on treatment of non-tuberculous mycobacterial pulmonary disease? Indian J. Med. Res. 154, 405–409. doi: 10.4103/ijmr.ijmr_2573_21, PMID: 35345066 PMC9131807

[B120] Shenai S. Rodrigues C. Mehta A. (2009). Rapid speciation of 15 clinically relevant mycobacteria with simultaneous detection of resistance to rifampin, isoniazid, and streptomycin in Mycobacterium tuberculosis complex. Int. J. Infect. Dis. 13, 46–58. doi: 10.1016/j.ijid.2008.03.025, PMID: 18565777

[B121] Shenai S. Rodrigues C. Mehta A. (2010). Time to identify and define non-tuberculous mycobacteria in a tuberculosis-endemic region. Int. J. Tuberc Lung Dis. 14, 1001–1008., PMID: 20626945

[B122] Shrivastava K. Kumar C. Singh A. Narang A. Giri A. Sharma N. K. . (2020). An overview of pulmonary infections due to rapidly growing mycobacteria in South Asia and impressions from a subtropical region. Int. J. Mycobacteriol 9, 62–70. doi: 10.4103/ijmy.ijmy_179_19, PMID: 32474491

[B123] Simoes E. A. Babu P. G. John T. J. Nirmala S. Solomon S. Lakshminarayana C. S. . (1987). Evidence for HTLV-III infection in prostitutes in Tamil Nadu (India). Indian J. Med. Res. 85, 335–338., PMID: 3623641

[B124] Singh S. Gopinath K. Shahdad S. Kaur M. Singh B. Sharma P . (2007). Nontuberculous mycobacterial infections in Indian AIDS patients detected by a novel set of ESAT-6 polymerase chain reaction primers. Jpn J. Infect. Dis. 60, 14–18. doi: 10.7883/yoken.JJID.2007.14, PMID: 17314419

[B125] Singh K. Kumari R. Tripathi R. Gupta S. Anupurba S . (2020). Detection of clinically important non tuberculous mycobacteria (NTM) from pulmonary samples through one-step multiplex PCR assay. BMC Microbiol. 20, 267. doi: 10.1186/s12866-020-01952-y, PMID: 32847517 PMC7448335

[B126] Joint Position Paper of the American Thoracic Society and the Centers for Disease Control . (1987). Mycobacterioses and the acquired immunodeficiency. Am. Rev. Respir. Dis. 136, 492–496. doi: 10.1164/ajrccm/136.2.492, PMID: 3304048

[B127] Steffen U. Koeleman C. A. Sokolova M. V. Bang H. Kleyer A. Rech J. . (2020). IgA subclasses have different effector functions associated with distinct glycosylation profiles. Nat. Commun. 11, 120. doi: 10.1038/s41467-019-13992-8, PMID: 31913287 PMC6949214

[B128] Subcommittee of the Joint Tuberculosis Committee of the British Thoracic Society. Management of opportunist mycobacterial infections: Joint Tuberculosis Committee Guidelines 1999. Thorax 55, 210–218. 10.1136/thorax.55.3.210 PMC1745689 10679540

[B129] Sun Z. Zhang Y. (1999). Reduced pyrazinamidase activity and the natural resistance of *Mycobacterium kansasii* to the antituberculosis drug pyrazinamide. Antimicrob. Agents Chemother. 43, 537–542. doi: 10.1128/AAC.43.3.537, PMID: 10049264 PMC89157

[B130] Szulga T. Jenkins P. A. Marks J. (1966). Thin-layer chromatography of mycobacterial lipids as an aid to classification; *Mycobacterium kansasii*; and *Mycobacterium marinum* (balnei). Tubercle 47, 130–136. doi: 10.1016/S0041-3879(66)80055-7, PMID: 5936896

[B131] Tarshis M. S. Frisch A. W. (1952a). Chromogenic acid-fast bacilli from human sources. I. Cultural studies. Am. Rev. Tuberc 65, 278–288. doi: 10.1164/art.1952.65.3.278, PMID: 14903495

[B132] Tarshis M. S. Frisch A. W. (1952b). Chromogenic acid-fast bacilli from human sources. II. Pathologic studies. Am. Rev. Tuberc 65, 289–301. doi: 10.1164/art.1952.65.3.289, PMID: 14903496

[B133] Tarshis M. S. Frisch A. W. (1952c). Chromogenic acid-fast bacilli from human sources. III. Hypersensitivity Stud. Am. Rev. Tuberc 65, 302–315. 10.1164/art.1952.65.3.302 14903497

[B134] Thangavelu K. Krishnakumariamma K. Pallam G. Dharm Prakash D. Chandrashekar L. Kalaiarasan E. . (2021). Prevalence and speciation of non-tuberculous mycobacteria among pulmonary and extrapulmonary tuberculosis suspects in South India. J. Infect. Public Health 14, 320–323. doi: 10.1016/j.jiph.2020.12.027, PMID: 33618276

[B135] Thompson R. N. Blumenthal A. Morrison M. Thomson R. M. (2025). The microbiome and gut–lung axis in nontuberculous mycobacterial pulmonary disease. PloS Pathog. 21, e1013603. doi: 10.1371/journal.ppat.1013603, PMID: 41105671 PMC12533858

[B136] Timpe A. Runyon E. H. (1954). The relationship of atypical acid-fast bacteria to human disease; a preliminary report. J. Lab. Clin. Med. 44, 202–209. 13184228

[B137] Torrelles J. B. Ellis D. Osborne T. Hoefer A. Orme I. M. Chatterjee D. . (2002). Characterisation of virulence, colony morphotype and the glycopeptidolipid of *Mycobacterium avium* strain 104. Tuberculosis (Edinb) 82, 293–300. doi: 10.1054/tube.2002.0373, PMID: 12623272

[B138] Tsukamura M. (1967). Identification of mycobacteria. Tubercle 48, 311–338. doi: 10.1016/S0041-3879(67)80040-0, PMID: 4968513

[B139] Turenne C. Y. (2019). Nontuberculous mycobacteria: Insights on taxonomy and evolution. Infect. Genet. Evol. 72, 159–168. doi: 10.1016/j.meegid.2019.01.017, PMID: 30654178

[B140] Vise E. Das S. Garg A. Karam A. Ghatak S. Sen A. . (2016). Isolation and identification of a novel Non-tuberculous Mycobacterium species of canine origin by multiple gene sequencing approach. Int. J. Infect. Dis. 45, 414–415. doi: 10.1016/j.ijid.2016.02.884

[B141] Wallace R. J. Jr Meier A. Brown B. A. Zhang Y. Sander P. Onyi G. O. . (1996). Genetic basis for clarithromycin resistance among isolates of *mycobacterium chelonae* and *Mycobacterium abscessus*. Antimicrob. Agents Chemother. 407, 1676–1681. doi: 10.1128/AAC.40.7.1676, PMID: 8807061 PMC163394

[B142] Wani S. R. Wattal C. Raveendran R. (2020). Epidemiology and risk factors associated with NTM pulmonary and extrapulmonary infections in a high tuberculosis endemic Region. Indian J. Med. Microbiol. 38, 169–175. doi: 10.4103/ijmm.IJMM_20_274, PMID: 32883930

[B143] Watanabe M. Banno S. Sasaki K. Naniwa T. Hayami Y. Ueda R . (2011). Serodiagnosis of *Mycobacterium avium*-complex pulmonary disease with an enzyme immunoassay kit that detects anti-glycopeptidolipid core antigen IgA antibodies in patients with rheumatoid arthritis. Mod Rheumatol 21, 144–149. doi: 10.1007/s10165-010-0368-5, PMID: 21082209

[B144] Wayne L. G. (1966). Classification and identification of mycobacteria. 3. Species within group 3. Am. Rev. Respir. Dis. 93, 919–928. doi: 10.1164/arrd.1966.93.6.919, PMID: 5942239

[B145] Wayne L. G. (1988). International Committee on Systematic Bacteriology: announcement of the report of the Adhoc Committee on Reconciliation of Approaches to Bacterial Systematics. Zentralbl Bakteriol Mikrobiol Hyg A. 268, 433–434. doi: 10.1016/s0176-6724(88)80120-2, PMID: 3213314

[B146] Wazahat R. Juneja P. Chauhan V. Zaidi R. Lamichhane G. Singh U. B. . (2023). A strip-based assay for detection of CrfA enzyme activity to differentiate Mycobacterium tuberculosis and non-tuberculous mycobacteria. J. Microbiol. Methods 211, 106781. doi: 10.1016/j.mimet.2023.106781, PMID: 37437716

[B147] Wilson G. S. (1925). Serological classification of the tubercle bacilli by agglutination and absorption of agglutinins. J. Pathol. Bacteriol 28, 69–96. doi: 10.1002/path.1700280105

[B148] Wolinsky E. (1979). Nontuberculous mycobacteria and associated diseases. Am. Rev. Respir. Dis. 119, 107–159. doi: 10.1164/arrd.1979.119.1.107, PMID: 369415

[B149] Wolinsky E. (1981). When is an infection disease? Rev. Infect. Dis. 3, 1025–1027. 7339799 10.1093/clinids/3.5.1025

[B150] Wolinsky E. Smith M. M. Mitchell R. S. Steenken W. Jr (1957). Atypical chromogenic mycobacteria associated with pulmonary disease; including a report of three cases. Am. Rev. Tuberc 75, 180–198. doi: 10.1164/artpd.1957.75.2.180, PMID: 13403151

[B151] Woods G. L. Washington J. A. (1987). Mycobacteria other than Mycobacterium tuberculosis: review of microbiologic and clinical aspects. Rev. Infect. Dis. 9, 275–294. doi: 10.1093/clinids/9.2.275, PMID: 3296098

[B152] Wu U. I. Holland S. M. (2015). Host susceptibility to non-tuberculous mycobacterial infections. Lancet Infect. Dis. 15, 968–980. doi: 10.1016/S1473-3099(15)00089-4, PMID: 26049967

[B153] Yoder W. D. Schaefer W. B. (1971). Comparison of the seroagglutination test with the pathogenicity test in the chicken for the identification of *Mycobacterium avium* and Mycobacterium intracellulare. Am. Rev. Respir. Dis. 103, 173–178., PMID: 5541465 10.1164/arrd.1971.103.2.173

[B154] Young L. S. Inderlied C. B. Berlin O. G. Gottlieb M. S Gottlieb M. S . (1986). Mycobacterial infections in AIDS patients, with an emphasis on the *Mycobacterium avium* complex. Rev. Infect. Dis. 8, 1024–1033. doi: 10.1093/clinids/8.6.1024, PMID: 3541122

[B155] Young L. S. Wiviott L. Wu M. Kolonoski P. Bolan R. Inderlied C.B . (1991). Azithromycin for treatment of *Mycobacterium avium*-intracellulare complex infection in patients with AIDS. Lancet 338, 1107–1109. doi: 10.1016/0140-6736(91)91965-W, PMID: 1682544

[B156] Yuste J. R. Bertó J. del Pozo J. L. Leiva J . (2017). Prolonged use of tedizolid in a pulmonary non-tuberculous mycobacterial infection after linezolid-induced toxicity. J. Antimicrob. Chemother. 72, 625–628. doi: 10.1093/jac/dkw484, PMID: 27999019

