# Supplementary material for: From anonymity to stardom: history of nontuberculous mycobacterial disease in humans
Source: Front Cell Infect Microbiol. 2026 Feb 23;15:1717909. doi: 10.3389/fcimb.2025.1717909 (PMC12968181; doi:10.3389/fcimb.2025.1717909)
Supplement: Supplementary file 1 [file Table1.docx]

| **Supplementary table 1**: Cardinal differences between nontuberculous mycobacteria and *Mycobacterium tuberculosis* | | | |
| --- | --- | --- | --- |
| **S.no.** | **Characteristics** | **Nontuberculous mycobacteria (NTM)** | ***Mycobacterium tuberculosis* (MTB)** |
|  | **Nomenclature** | - Preferred name: nontuberculous mycobacteria (NTM) - Other names: mycobacteria other than tuberculosis (MOTT), atypical mycobacteria, anonymous mycobacteria, and environmental mycobacteria | - *Mycobacterium tuberculosis* (*Mtb*): member of *Mycobacterium tuberculosis* complex (MTBC) - Other species in MTBC: *M. africanum*, *M. bovis*, *M. canetti, M. caprae and M. pinnipedii* |
|  | **NTM species distribution** | Nearly 200 valid species. Regional variation of NTM species due to climatic and geographical factors | *Mtb* strains: Beijing (most pathogenic), Cameroon, CAS, EAI, Haarlem, LAM, Manu (Indian) |
|  | **Biochemical tests** | No single biochemical test is available for the diagnosis of NTM species. Some of the NTM species show positive results with niacin accumulation test (*M. simiae, M. chelonae*), nitrate reduction test (*M. ulcerans, M. szulgai, M. fortuitum, M. smegmatis, M. kansasii*)*,* catalase test (*M. fortuitum, M. chelonae, M. abscessus, M. ulcerans, M. szulgai, M. kansasii*)*,* citrate utilization test (*M. chelonae, M. smegmatis*), urea hydrolysis test (*M. kansasii, M. marinum, M. simiae, M. szulgai, M. scrofulaceum* ), McConkey agar (without crystal violet) (*M. fortuitum, M. abscessus* ) test and tellurite reduction (*M. avium, M. intracellulare, M. simiae, M. fortuitum, M. abscessus*) | *Mtb* is niacin positive, reduces nitrate, and is negative for heat-stable catalase test |
|  | **Microscopic morphology** | Absence of characteristic serpentine cords in acid-fast smears | - Characteristic serpentine cording seen as rope-like aggregates in which long axis of the bacilli is parallel to the long axis of the cord in acid-fast smears - *Mtb* colonies are rough, cauliflower-like, and light buff in colour |
|  | **Growth characteristics**  **in cultures** | - Rapidly growing mycobacteria (<7days) Slowly growing mycobacteria (≥7days) - Growth rates of NTM are slower than other bacteria (*Pseudomonas aeruginosa and E. coli*) | - *Mtb* are slowly growing mycobacteria and take ~ 2 weeks to grow - Ordinary bacteria may take ~ 20 mins to 12-24 hr in the laboratory |
|  | **Differential identification** | - Difficult to differentiate NTM from *Mtb* only on the basis of positive acid-fast smear - Culture is important in differentiating from *Pseudomonas aeruginosa*, *Staphylococcus aureus*, Nocardia, Aspergillus, and Sporothrix, etc. | Both smear and culture should be done |
|  | **Transmission** | Person-to-person transmission does not occur except for *M. abscessus* among cystic fibrosis patients | *Mtb* is highly transmissible, *especially* in pulmonary tuberculosis with cavitary disease and high bacillary loads |
|  | **Route of entry** | - Sources: inhalation, ingestion, or direct inoculation. - Airborne NTM are a major source of entry for NTM pulmonary disease (NTM-PD). - In advanced HIV/AIDS, gut colonization with subsequent haematogenous dissemination occurs | Smaller cough droplet nuclei (<1-10 µM) carrying *Mtb* reach terminal bronchioles and alveoli and establish infection |
|  | **Pathogenicity potential** | Opportunistic organisms | Highly pathogenic and obligate parasites |
|  | **Virulence** | - Generally, NTM have low virulence - *M. kansasii* is more virulent among NTM | Highly virulent |
|  | **Latent infection** | No evidence of latent NTM infection | - Systematic data are available regarding latent TB infection (LTBI), especially in low TB-burden countries - Latent TB infection is an important source of future active disease |
|  | **Case notification and surveillance** | - It is not essential to notify laboratory-confirmed, newly diagnosed NTM cases - NTM disease notification and surveillance are practiced only in a few high-income countries | - Systematic TB notification and periodic surveillance are encouraged in most countries - The global tuberculosis report is published annually on a regular basis by the World Health Organization |
|  | **Pulmonary: extrapulmonary disease proportions** | - Pulmonary: extrapulmonary 80%-90%: 10%-20% in HIV-negative - Disseminated NTM disease occurs in severely immunocompromised individuals, such as those with advanced HIV/AIDS | - Pulmonary 80-85%: extrapulmonary 15-20 % in HIV-negative patients - Pulmonary 40-50%: extrapulmonary 50%-60% in HIV/AIDS |
|  | **Risk factors** | Risk factors for NTM-PD:   - Pre-existing lung disease - Primary ciliary dyskinesia - Bronchiectasis* - Impaired muco-ciliary function or in individuals who are heterozygous for CFTR mutations - Gastroesophageal reflux disease (GERD)**†** | TB can involve both healthy and destroyed lungs  Risk factors include: malnutrition, tobacco smoking, chronic alcohol intake, diabetes mellitus, overcrowding, HIV/AIDS, head or neck cancer, leukaemia, or Hodgkin's disease, drugs including corticosteroids, TNF-α inhibitors, or receptor blockers |
|  | **NTM species predilection for various organs** | - **Pulmonary:** *M. avium* complex*, M. kansasii, M, xenopi, M. malmoense, M. abscessus, M. fortuitum M. simiae* - **Extrapulmonary**   *Skin*: *M. ulcerans, M. marinum, M. abscessus, M. chelonae, M. fortuitum*  *Soft tissues*: *M. abscessus, M. chelonae, M. chimaera*  *Lymphadenitis:* MAC but can occur with other NTM species also   - **Disseminated NTM disease**: most commonly due to MAC but other species can also produce disseminated disease | No such predilection for body organs is known in TB |
|  | **Radiographic patterns in pulmonary disease** | - Cavitary - Nodular/bronchiectatic (NB) - Hypersensitivity pneumonitis-like NTM-PD due to MAC**‡** | - Primary complex (usually in children) - Progressive pulmonary disease - Miliary pulmonary TB - Post-primary pulmonary TB: cavitary, atelectasis, consolidation - Sequelae such as fibrotic and calcified lesions |
|  | **Clinical relevance of NTM isolates in respiratory specimens** | - Clinical relevance of isolated NTM species versus activity of the underlying pulmonary disease should be assessed - Colonization in the host and contamination in the laboratory must be ruled out - Causality association of the particular isolated NTM species with the pulmonary disease should be carefully established before starting the treatment | - *Mtb* produces both latent TB infection and active disease - Active TB disease must be ruled out appropriately before starting the treatment |
|  | **Drug susceptibility testing (DST)** | - DST for NTM is controversial because of poor correlation between *in vitro* DST patterns and *in vivo* treatment response and outcomes - DST is performed according to CLSI (2023) guidelines - Both phenotypic and genotypic DST are performed - Phenotypic and genotypic DST for selected drugs:   MAC: macrolides (clarithromycin), amikacin   - *M. kansasii*: rifampicin, clarithromycin - RGM species: macrolides, amikacin, sulfamethoxazole, doxycycline, minocycline, tigecycline, cefoxitin, linezolid - Status of *erm* (41) gene and its function in *M. abscessus* subspecies should be known | - Universal DST should be performed, and treatment should be carried out as per the sensitivity profile of *Mtb* - As per the sensitivity profile of *Mtb*, disease should be categorised into DS-TB, Hr-TB, MDR/RR-TB, and XDR-TB |
|  | **Treatment** | Various guidelines for treatment of NTM diseases   - American Thoracic Society (ATS), 2007 - British Thoracic Society (BTS), 2017   ATS/ERS/ESMID/IDSA, 2020 | National guidelines should be followed for treatment of drug-sensitive and DR-TB |
|  | **Treatment outcomes** | Treatment outcomes differ among NTM species and subspecies, and host characteristics | - Globally, treatment outcomes in case of drug-sensitive TB are good - Treatment of drug-resistant TB is still a challenge, and global rate of successful treatment is 63% only - With newer drug regimen(s), treatment success rates are likely to improve in future |
|  | **Prevention** | - Exposure to NTM from environmental sources, *especially* household water systems, hospital settings, and soil, should be avoided - In HIV/AIDS patients (CD4 T-cells counts<50/μL), antimicrobial prophylaxis includes administration of azithromycin (1200mg/weekly) or clarithromycin (500mg twice daily) or rifabutin (300mg/day) along with antiretroviral drugs till CD4 cell count is >100 cells/ μL for 3 months | - Exposure to smear-positive pulmonary TB should be avoided to halt TB transmission - Chemoprophylaxis for latent TB infection (active TB disease must be ruled out in high TB-burden countries), various treatment options include: isoniazid daily for 6 or 9 months. or a combination of rifapentine and isoniazid once weekly for 12 weeks or the combination of rifampicin and isoniazid daily for 3-4 months, or rifampicin alone daily for 4 months |
|  | **Vaccines** | No vaccine is available at present because of several species | BCG vaccine is recommended in high TB burden countries to prevent severe forms of TB (miliary and central nervous system TB); newer TB vaccines such as M72/AS01, *M. vaccae*, MVA85A, etc, are in clinical trials. M72/AS01 was significantly protective against TB disease in a Phase IIb trial in Kenya |

NTM= nontuberculous mycobacteria; CAS=Central Asian strain; EAI=East African Indian strain; LAM=Latin American-Mediterranean strain; CFTR= cystic fibrosis transmembrane regulator gene; PTB=pulmonary TB; GERD: gastroesophageal reflux disorder; COPD= chronic obstructive pulmonary disease; HIV/AIDS = human immunodeficiency virus/acquired immunodeficiency syndrome; TNF-α=tumour necrosis factor-α; SGM = slowly growing mycobacteria; RGM= rapidly growing mycobacteria; DS-TB=drug sensitive-TB; Hr-TB= isoniazid resistant-TB; MDR/RR-TB=multidrug resistant/rifampicin resistant-TB; XDR-TB= Extensively drug-resistant TB; ERS= European Respiratory society; ESMID= European Society of Clinical Microbiology and Infectious Diseases; IDSA=Infectious Disease Society of America; *erm* = erythromycin ribosome methylation.

*In low TB-endemic countries causes of bronchiectasis are other than TB (cystic fibrosis, COPD, etc) whereas in high TB-burden countries post-TB bronchiectasis is common

**†**Underlying oesophageal disease must be ruled out in NTM-PD due to RGM, especially *M. fortuitum.* Patients with HIV/AIDS

**‡**Rare in India

**Source:** Table 3 from Sharma SK, Upadhyay V. Epidemiology, diagnosis & treatment of non-tuberculous mycobacterial diseases. Indian J Med Res. 2020 Sep;152(3):185-226 *(reference 66)* is reproduced from the Indian Journal of Medical Research; copyright of Indian Council of Medical Research, New Delhi, with permission.
